# Siah2 regulates lipid uptake in adipose tissue macrophages

**DOI:** 10.1016/j.jbc.2026.111380

**Published:** 2026-03-18

**Authors:** Bhaswati Ghosh, Pradip R. Panta, Matthew C. Scott, Jessica Taylor, Robbie Beyl, Krisztian Stadler, Z. Elizabeth Floyd

**Affiliations:** Pennington Biomedical Research Center, Baton Rouge, Louisiana, USA

**Keywords:** adipose tissue macrophages, Siah2, PPARγ, CD36, ubiquitin ligase, lysosome, lipid metabolism, lipolysis, inflammation

## Abstract

In obesity, adipose tissue (AT) macrophages (ATMs) reprogram their metabolism to influence AT remodeling and function. Ubiquitin (Ub) ligases are critical in modulating the degradation of key proteins implicated in macrophage lipid metabolism. Yet, the role of Ub ligases in ATM lipid metabolism is largely unexplored. Previously, we reported that the Ub ligase Siah2 (seven in absentia homolog 2) is crucial in mediating adipogenic pathways and AT inflammation. Here, we cocultured bone marrow–derived macrophages with AT as an *ex vivo* model of bone marrow–derived ATMs to investigate the role of Siah2 in ATM lipid metabolism. We found that AT-induced lipid accumulation in ATMs was exacerbated by Siah2 deficiency *via* increased CD36-mediated lipid uptake and reduced lipid delivery to lysosomes. Together, these changes contributed to excessive lipid accumulation, lipid peroxidation, and an inflammatory phenotype. Our data reveal a central role for Siah2 as a lipid uptake sensor in maintaining the balance between lipid influx and degradation in ATMs.

Obesity is a disease of excessive fat deposition that leads to metabolic consequences, including cardiovascular diseases, disrupted insulin signaling, type 2 diabetes, and musculoskeletal disorders (1, 2, 3). Evidence is accumulating that adipose tissue (AT) inflammation, coupled with lipid deposition in adipose tissue macrophage (ATM), is linked with obesity-induced insulin resistance (4, 5, 6). The adverse relationship between lipid-laden ATMs and insulin resistance has increased interest in understanding how ATMs process lipids in AT. Although the ATMs are remarkably capable of switching phenotypes as a homeostatic response to excess lipid accumulation (7, 8, 9), prolonged lipid accumulation in ATMs can be proinflammatory (5). But despite growing evidence that lipid accumulation in ATMs contributes to obesity-related insulin resistance, the molecular mechanisms underlying ATM lipid handling in response to the metabolic challenge of excess lipid exposure are not well understood.

The ubiquitin (Ub)–proteasome system (UPS) is a central regulator of protein homeostasis. Decades of research establish that UPS governs the stability of key players implicated in macrophage lipid metabolism (10). Of the more than 600 E3 Ub ligases in the human genome, several play diverse roles in the regulation of lipid synthesis, lipid transport, and lipid degradation in macrophages (11, 12) and AT inflammation (13). However, the role of E3 Ub ligases in ATM lipid metabolism is largely unexplored.

The RING-type E3 Ub ligase seven in absentia homolog 2 (Siah2) is well characterized as regulating cellular stress responses. Under hypoxic conditions, Siah2 stabilizes hypoxia-inducible factor-1α to promote hypoxia-inducible factor-1α–mediated responses to hypoxia (14). In response to cytokine signaling and DNA damage, Siah2 targets tumor necrosis factor (TNF) receptor–associated factor 2 (TRAF2) for degradation and induces apoptosis by inhibiting the antiapoptotic effects of TRAF2 (15). Moreover, Siah2-mediated TRAF2 degradation attenuates activation of inflammatory signals related to c-Jun N-terminal kinase and NF-κB pathways (15). We previously reported that Siah2 is upregulated and interacts with the lipid-activated transcription factor peroxisome proliferator–activated receptor gamma (PPARγ) during adipogenesis (16) and is crucial for regulating the early events in adipogenesis (17, 18). Moreover, global deletion of Siah2 increases expression of a subset of PPARγ target genes and proteins involved with lipid metabolism and inflammatory signaling (19). The metabolic consequences of Siah2 loss appear to be cell type dependent. *Siah2* mRNA is widely expressed in AT immune cells and upregulated in ATMs from male mice with obesity. Unlike global Siah2 deletion, macrophage-specific depletion of Siah2 leads to glucose intolerance and insulin resistance in an animal model of obesity (20). The changes in metabolic function are associated with increased AT inflammation and increased lipid accumulation in the Siah2-deficient ATM. Based on these results and the presence of *Siah2* mRNA in macrophages surrounding dying adipocytes (18), we hypothesized that Siah2 in ATMs is involved in regulating lipid handling and inflammation in ATMs. To assess the role of Siah2 in ATM lipid accumulation and inflammation in response to lipid challenges, we used macrophage-specific Siah2-deficient bone marrow–derived macrophages (BMDMs) in an *ex vivo* model of ATMs developed by Xu *et al*. (21). With this approach, we identified Siah2 as an essential sensor of lipid accumulation in ATMs. This provides mechanistic insights into how a Ub ligase regulates the dynamic relationship between lipid handling and phenotypic adaptation to lipid challenges in ATM.

## Results

### Macrophage-specific Siah2 knockout leads to robust lipid accumulation in ATMs

**Figure 1:**
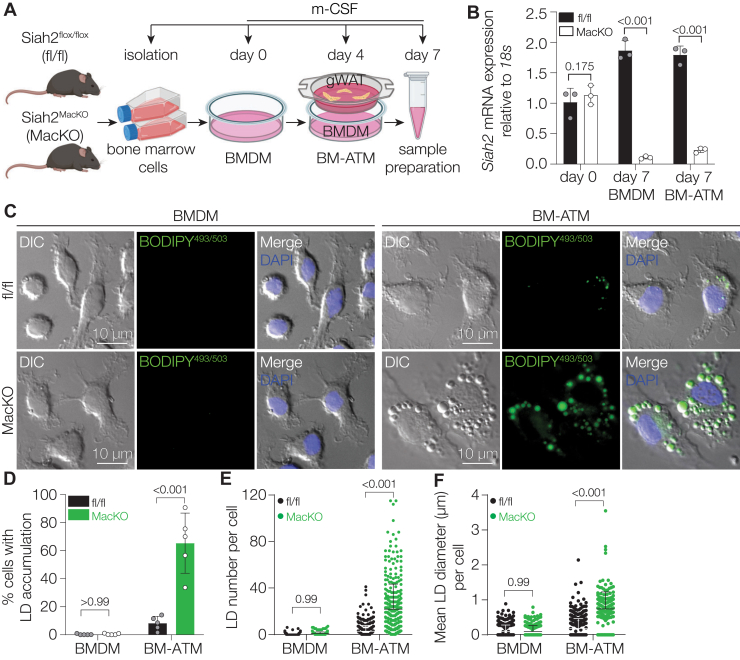
**Macrophage-specific Siah2 depletion leads to excessive lipid accumulation in ATMs**. *A*, schematic illustrating the protocol for differentiation of mouse BMDMs into BM-ATMs in the presence of gWAT derived from lean wildtype mice. *B*, relative mRNA expression of *Siah2* in bone marrow cells (day 0), BMDMs, and BM-ATMs derived from fl/fl and *Siah2*^MacKO^ mice. *C*, representative DIC, fluorescence (LD, *green*; nuclei, *blue*) and overlaid images of BMDMs and BM-ATMs derived from fl/fl and *Siah2*^MacKO^ female mice. The scale bar represents 10 μm. *D*–*F*, quantification of *C*. LD number per cell (*E*) and mean LD diameter (μm) per cell (*F*) in BMDMs and BM-ATMs, derived from fl/fl and *Siah2*^MacKO^. Statistics are reported as mean ± SD using an unpaired *t* test with Welch’s correction. *N* = 4 mice per group. *P* Values are indicated on the graphs. ATM, adipose tissue macrophage; BM-ATM, bone marrow–derived ATM; BMDM, bone marrow–derived macrophage; DIC, differential interference contrast; fl/fl, *Siah2*^flox/flox^; gWAT, gonadal white adipose tissue; LD, lipid droplet; MacKO, *Siah2*^MacKO^; Siah2, seven in absentia homolog 2.

To study ATMs, we adopted the *ex vivo* model of bone marrow–derived ATM (BM-ATM) described in the study by Xu *et al*. (21). This model differentiates bone marrow–derived cells with macrophage-colony stimulating factor in the presence or the absence of gonadal white adipose tissue (gWAT) to obtain BMDM or BM-ATM (Fig. 1*A*). To investigate the role of Siah2 in macrophage lipid handling in unactivated BMDM and BM-ATMs, we used *Siah2*^flox/flox^ (hereafter, *Siah2*^flox/flox^ will be referred to as fl/fl) and *Siah2*^MacKO^ (macrophage-specific *Siah2* knockout) mouse lines. We first confirmed *Siah2* depletion in BMDMs and BM-ATMs by RT–PCR (Fig. 1*B*). Because Siah2 is necessary for preadipocyte differentiation, we then sought to determine if SIAH2 deficiency impacts macrophage differentiation. At day 7 postinduction of unactivated BMDMs, expression of monocyte markers (*Ccr2*, *Ly6c*) was minimal, and canonical macrophage markers (*Cd64*, *Cd68*, and *F4/80*) were upregulated equally in fl/fl and *Siah2*^MacKO^ BMDMs (Fig. S1*A*). This demonstrates that macrophage differentiation is unaffected by Siah2 depletion. However, both *Siah2*^MacKO^ BMDMs and BM-ATMs were phenotypically different from fl/fl (Fig. 1*C*). We observed that macrophage-specific Siah2 loss resulted in robust lipid accumulation in BM-ATMs (Fig. 1*C* and S1*B*). BODIPY^493/503^-positive lipid droplets (LDs) in the *Siah2*^MacKO^ BM-ATMs were significantly more abundant and larger compared with fl/fl BM-ATMs (Fig. 1, *D*–*F*). Consistently, increased LD accumulation was observed in F4/80-expressing *Siah2*^MacKO^ BM-ATMs compared with fl/fl BM-ATMs (Fig. S1*B*). These data indicate that Siah2’s function is necessary to restrict lipid accumulation in ATMs when exposed to an AT challenge.

### Macrophage-specific Siah2 deficiency induces a distinct proinflammatory switch in ATMs

**Figure 2:**
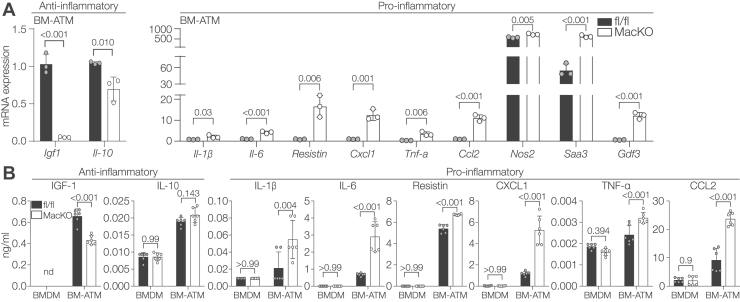
**Macrophage-specific Siah2 loss induces an anti- to-proinflammatory switch in ATMs**. *A*, relative mRNA expression of anti-inflammatory and proinflammatory genes in fl/fl and *Siah2*^MacKO^ BM-ATMs. *B*, concentration (ng/ml) of anti-inflammatory and proinflammatory factors, released in the conditioned media of fl/fl and *Siah2*^MacKO^ BMDMs and BM-ATMs, measured by Luminex assay. Statistics are reported as mean ± SD using an unpaired *t* test with Welch’s correction. Each *dot* represents a technical replicate. Data are representative of two independent experiments. *n* = 3 male mice per group. *p* Values are indicated on the graphs. ATM, adipose tissue macrophage; BM-ATM, bone marrow–derived ATM; BMDM, bone marrow–derived macrophage; fl/fl, *Siah2*^flox/flox^; MacKO, *Siah2*^MacKO^; Siah2, seven in absentia homolog 2.

Lipid accumulation is strongly correlated with proinflammatory responses. In obese AT, elevated fatty acid accumulation contributes to macrophage activation and a proinflammatory phenotype (22). Since we previously demonstrated that Siah2 regulates obesity-induced inflammation in mouse AT (19), we hypothesized that Siah2 plays a role in modulating inflammation in ATMs during lipid exposure. We tested anti-inflammatory markers (interleukin [*Il*]*-10*, *Igf1*) (23, 24); classical proinflammatory signatures (*Il-1b*, *Il-6*, *Tnf-a*, *Nos2*, and *Gdf3*) (23, 25), chemokines (*Cxcl1*, *Ccl2*) (23), a chemokine-like factor (*Saa3*) (26), and *resistin*, an adipokine that links obesity to inflammation (27). We found that Siah2 deficiency in BM-ATMs induces a distinct proinflammatory profile by decreasing gene expression of anti-inflammatory targets and increasing proinflammatory targets (Fig. 2*A*). Full gene names are provided in Table S1.

Macrophages are reported to be the primary source of released inflammatory cytokines and chemokines in obesity and atherosclerosis mouse models (28, 29). For this reason, we asked if the ATM secretome is altered by Siah2 deficiency. We used a customized Luminex assay kit (R&D Systems) and the conditioned media derived from fl/fl and *Siah2*^MacKO^ BMDMs and BM-ATMs to measure the concentration of secreted anti-inflammatory and proinflammatory factors that were assayed for gene expression (except NOS-2, SAA-3, and GDF-3). The levels of all tested analytes were increased when gWAT was present in the BM-ATMs compared with the BMDMs (Fig. 2*B*). Furthermore, macrophage-specific Siah2 depletion in BM-ATMs significantly decreased (insulin-like growth factor 1) or did not change (IL-10) the production of anti-inflammatory markers but increased secretion of the proinflammatory cytokines, IL-1β, IL-6, resistin, CXCL1, TNF-α, and CCL2 (Fig. 2*B*). Taken together, these data indicate that Siah2 depletion in BM-ATMs triggers an intracellular inflammatory switch that induces the release of proinflammatory factors in the extracellular milieu.

Because inflammation is known to induce lipolysis in AT (30), we asked if the proinflammatory response in the Siah2-deficient ATMs affected AT lipolysis. To test this, we incubated freshly isolated gWAT with control media or conditioned media from BM-ATMs and measured AT lipolysis (Fig. S2). Lipolysis was unchanged by conditioned media from the fl/fl BM-ATMs, but free fatty acid and glycerol release from the AT were increased when exposed to the conditioned media obtained from the *Siah2*^MacKO^ BM-ATM (Fig. S2, *A* and *B*). Thus, AT lipolysis was stimulated by the proinflammatory factors secreted by Siah2-deficient BM-ATMs.

### Siah2 regulates lipid peroxidation in ATMs

**Figure 3:**
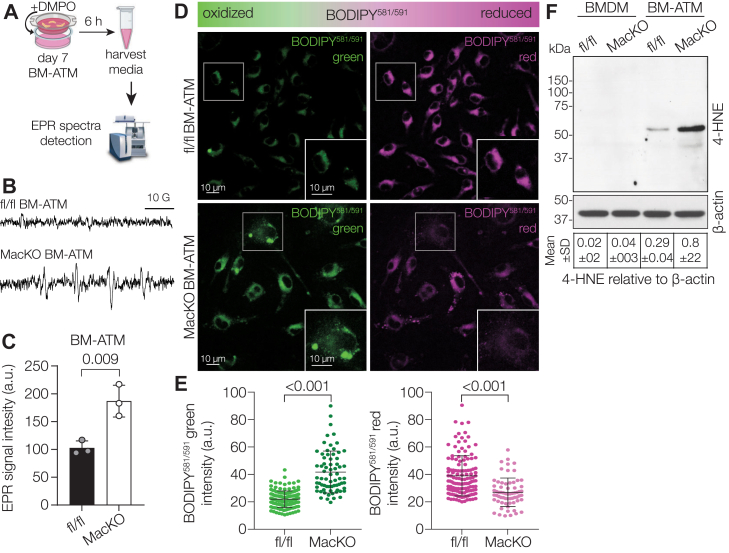
**Macrophage-specific Siah2 deficiency results in lipid peroxidation in ATMs**. *A*, schematic illustrating the protocol for superoxide production assay in BM-ATMs. *B*, four-line EPR spectra of DMPO-lipid radical adducts measured in fl/fl and *Siah2*^MacKO^ BM-ATM conditioned media. *C*, quantification of *B*. Bar graph showing EPR signal intensities (arbitrary units) of the measured peak amplitudes. *D*, representative fluorescence microscopy images of fl/fl and *Siah2*^MacKO^ BM-ATMs stained for lipid peroxidation sensor BODIPY^581/591^ (oxidized lipid, *green*; reduced lipid, *magenta*). The scale bar represents 10 μm. *Insets* represent the boxed region, and the scale bar represents 10 μm. *Red signals* were converted to *magenta* using the LUT function in ImageJ. *E*, quantification of *D*. Mean fluorescence intensity of green signal and red signal of BODIPY^581/591^ in fl/fl and *Siah2*^MacKO^ BM-ATMs. *F*, Western blot and quantification of 4-HNE-modified proteins relative to β-actin in fl/fl and *Siah2*^MacKO^ BMDMs and BM-ATMs. Statistics are reported as mean ± SD using the unpaired *t* test with Welch’s correction. *n* = 2 to 3 male mice per group. *p* Values are indicated on the graphs. 4-HNE, 4-hydroxynonenal; ATM, adipose tissue macrophage; BM-ATM, bone marrow–derived ATM; BMDM, bone marrow–derived macrophage; DMPO, 5,5-dimethyl-1-pyrroline-N-oxide; EPR, electron paramagnetic resonance; fl/fl, *Siah2*^flox/flox^; MacKO, *Siah2*^MacKO^; Siah2, seven in absentia homolog 2.

In chronic inflammatory diseases, inflammation is often accompanied by lipid peroxidation, a process in which reactive oxygen species–modified lipids result in damaged cell membranes, oxidative stress, and cell death (31). In obesity, ATMs release proinflammatory cytokines that are associated with lipid peroxidation (32). Based on our data showing a significant proinflammatory switch (Fig. 2), coupled with increased lipid accumulation (Fig. 1) in response to Siah2 deficiency in BM-ATMs, we predicted increased lipid peroxidation in the *Siah2*^MacKO^ BM-ATMs. To test this prediction, we first assessed if an oxidative state conducive to initiating lipid peroxidation occurs in the *Siah2*^MacKO^ BM-ATMs. Using electron paramagnetic resonance (EPR) spectroscopy to measure superoxide production in the BM-ATM culture media (Fig. 3*A*), we found that superoxide production is significantly increased in the *Siah2*^MacKO^ BM-ATMs, as shown in the EPR spectrum (Fig. 3*B*) and quantified signal intensity (Fig. 3*C*). We then used a lipid peroxidation sensor, BODIPY^581/591^, which shifts the fluorescence emission spectrum to red for reduced or green for oxidized lipids, to determine if lipid peroxidation occurs in the BM-ATMs. We found that peroxidized lipids (green fluorescence) were more prevalent in the *Siah2*^MacKO^ BM-ATMs, whereas the lipid species in the fl/fl BM-ATMs were predominantly in a reduced state (red fluorescence) (Fig. 3, *D* and *E*).

During the lipid peroxidation process, several reactive aldehydes are generated, among which 4-hydroxynonenal (4-HNE) is the most toxic and most frequently observed (33). 4-HNE is a highly reactive byproduct of lipid peroxidation that can form adducts with various cellular proteins that can be detected by multiple specific bands at different molecular weights on a western blot (34). Hence, we sought to determine the levels of 4-HNE-modified proteins as a measure of lipid peroxidation in response to the oxidative state present with macrophage-specific Siah2 loss. Despite the unexpected appearance of only one strongly expressed band, 4-HNE-mediated protein modification is substantially increased in Siah2-depleted BM-ATMs compared with fl/fl BM-ATMs (Fig. 3*F*). Together, the data indicate that the absence of Siah2 in BM-ATMs induces a metabolic shift of lipid state from reduced to oxidized and that Siah2 plays a central role in regulating the lipid peroxidation of intracellular lipids in the ATMs.

### Macrophage-specific Siah2 loss reprograms lipid metabolism by upregulating lipid metabolism genes

**Figure 4:**
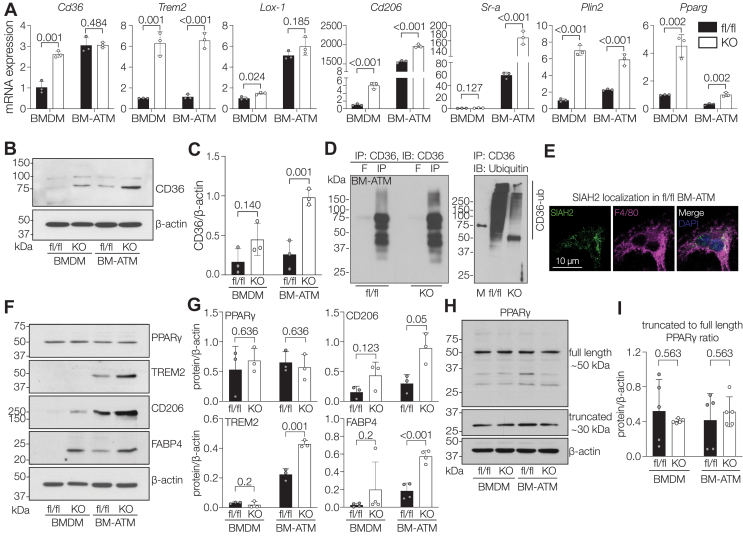
**Macrophage-specific Siah2 loss reprograms lipid metabolism in ATMs**. *A*, relative mRNA expression of lipid metabolism genes in fl/fl and *Siah2*^MacKO^ BMDMs and BM-ATMs. *B* and *C*, Western blot (*B*) and quantification (*C*) of CD36 protein levels relative to β-actin in fl/fl and *Siah2*^MacKO^ BMDMs and BM-ATMs. *D*, fl/fl and *Siah2*^MacKO^ BM-ATM whole cell extracts were harvested, and each whole cell extract (0.5 mg) was immunoprecipitated using anti-CD36 antibody and subjected to Western blot analyses using either anti-CD36 antibody (IP: CD36, IB: CD36) or antiubiquitin antibody (IP: CD36, IB: ubiquitin). Western blot images are representative of three independent experiments. *E*, fl/fl BM-ATMs immunolabeled for SIAH2 (*green*) and F4/80 (macrophage, *magenta*) and stained for DAPI (nuclei, *blue*). Red signals were converted to *magenta* using the LUT function in ImageJ. The *scale bar* represents 10 μm. *F* and *G*, Western blot (*D*) and quantification (*E*) of proteins involved with lipid uptake relative to β-actin in fl/fl and *Siah2*^MacKO^ BMDMs and BM-ATMs. *H*, Western blot of full-length and truncated PPARγ protein levels relative to β-actin in fl/fl and *Siah2*^MacKO^ BMDMs and BM-ATMs. *I*, ratio of truncated to full-length protein expression of PPARγ relative to β-actin in fl/fl and *Siah2*^MacKO^ BMDMs and BM-ATMs. Statistics are reported as mean ± SD using an unpaired *t* test with Welch’s correction. *n* = 3 to 4 male mice per group are representative of three independent experiments. *p* Values are indicated on the graphs. ATM, adipose tissue macrophage; BM-ATM, bone marrow–derived ATM; BMDM, bone marrow–derived macrophage; DAPI, 4′,6-diamidino-2-phenylindole; F, flow-through; fl/fl, *Siah2*^flox/flox^; IB, immunoblot; IP, immunoprecipitation; KO, *Siah2*^MacKO^; MacKO, *Siah2*^MacKO^; PPARγ, peroxisome proliferator–activated receptor gamma; Siah2, seven in absentia homolog 2.

Lipid accumulation can occur by increased lipid uptake (35, 36). Because macrophage-specific Siah2 depletion leads to excessive lipid accumulation in BM-ATMs (Fig. 1, *C*–*F* and Fig. S1*B*), we hypothesized that lipid uptake is a possible underlying mechanism. To test this possibility, we first analyzed the expression of genes involved with lipid uptake, including lipid scavenger receptors *Cd36* (cluster of differentiation 36), *Lox-1* (oxidized LDL receptor-1), *Cd206* (cluster of differentiation 206), and *Sr-a* (ccavenger receptor class A); lipoprotein receptor *Trem2* (triggering receptor expressed on myeloid cells 2); LD membrane protein *Plin2* (perilipin 2); and lipid-activated transcription factor *Pparg1*. We found that Siah2 depletion in macrophages markedly upregulated the lipid-associated genes we tested (Fig. 4*A*). Moreover, the elevated *Plin2* expression corresponded to increased abundance of PLIN2-positive LDs in *Siah2*^MacKO^ BM-ATMs compared with fl/fl BM-ATMs (Fig. S3, *A* and *B*). The fl/fl and *Siah2*^MacKO^ BM-ATMs also accumulated LDs that did not colocalize with PLIN2 (Fig. S3*C*)—a defining characteristic of BM-ATMs (37), confirming that our BM-ATM model recapitulates features of ATMs. In the absence of Siah2 in the BM-ATMs, the significant increase in perilipin-associated LD is more characteristic of perilipin-associated LDs found in foam cells (21). CD36 is a key lipid scavenging receptor involved with lipid uptake in macrophages (38). Notably, we observed that *Cd36* gene expression was increased in the BMDMs in response to macrophage-specific Siah2 deficiency and was further increased and saturated in the BM-ATMs for both genotypes (Fig. 4*A*). In contrast, immunoblots using whole cell lysate detected increased CD36 protein expression in Siah2-depleted BMDMs and BM-ATMs compared with fl/fl BMDMs and BM-ATMs (Fig. 4, *B* and *C*), suggesting Siah2-mediated post-translational regulation of CD36. To test if Siah2 affects Ub modification of CD36, we immunoprecipitated CD36 from fl/fl and *Siah2*^MacKO^ BM-ATMs and assayed CD36 and CD36 ubiquitylation patterns (Fig. 4*D*). Siah2 deficiency is associated with reduced ubiquitylation of CD36. This occurs although CD36 does not contain consensus Siah2 binding sites (39), consistent with an indirect effect of Siah2 in CD36 ubiquitylation. Although Siah2 is found in the BM-ATM nucleus when nuclear export is blocked (Fig. S3*E*), Siah2 shuttles between the nucleus and cytoplasm in response to signaling events (14, 40). We found Siah2 in the cytoplasmic and nuclear compartments of the fl/fl BM-ATMs (Fig. 4*E*), supporting a potential role of cytoplasmic Siah2 in regulating CD36 ubiquitylation and steady-state levels in macrophages.

We further evaluated the expression of additional proteins involved with lipid metabolism. TREM2, CD206, and FABP4 (fatty acid–binding protein 4) showed the highest increase in protein expression in Siah2-deficient BM-ATMs compared with BMDMs and fl/fl ATMs (Fig. 4, *F* and *G*). FABP4, a lipid chaperone that is crucial for lipid uptake (41), clearly showed a genotype-dependent increase in BMDMs and BM-ATMs. (Fig. 4, *F* and *G*). Ub modification of FABP4 shows a similar pattern to CD36, although the immunoisolated FABP4 also shifts to a higher molecular weight at 50 kD that is independent of Ub modification (Fig. S3*D*).

CD36 and FABP4 expressions are transcriptionally regulated by the nuclear receptor PPARγ, which plays a crucial role in macrophage functions, including differentiation, inflammatory response, and lipid metabolism (42). While PPARγ deficiency leads to reduced CD36 gene and protein expression in embryonic stem cell–derived macrophages (43), PPARγ activation upregulates CD36 (42). Because we show that PPARγ targets are upregulated in Siah2-depleted BM-ATMs (Fig. 4, *B*, *C*, *F*, *G*) and Siah2 regulates PPARγ ubiquitylation and degradation in adipocytes (16, 17, 19), we predicted that Siah2 depletion would elevate PPARγ1 protein levels as a mechanism underlying increased levels of PPARγ1 target proteins. However, we found no change in steady-state PPARγ1 protein expression in BMDMs and BM-ATMs across genotypes (Fig. 4, *F* and *G*). We further validated this result using two other antibodies: a monoclonal anti-PPARγ antibody that detected a truncated form (E-8; Santa Cruz Biotechnologies) and a polyclonal anti-PPARγ antibody (a kind gift from Dr Thomas W. Gettys) that detected both full-length (FL) and truncated forms of PPARγ1 protein in the macrophages (Fig. 4*H*). We then quantified the ratio of truncated to FL PPARγ1 and found no observable change across conditions and genotypes (Fig. 4*I*), suggesting that in our model, Siah2 deficiency upregulates PPARγ1 transcriptional activity and target protein levels independent of changes in PPARγ1 steady-state levels. Taken together, our data support Siah2-mediated regulation of a subset of PPARγ target genes and proteins associated with lipid uptake in ATMs. The data also support a role for Siah2 in post-translationally targeting the selected cytoplasmic proteins for Ub-mediated degradation, consistent with reduced Ub modification, increased steady-state levels of CD36 and FABP4, and cellular location of SIAH2 protein.

### Macrophage-specific Siah2 depletion elevates CD36-mediated lipid uptake in ATMs

**Figure 5:**
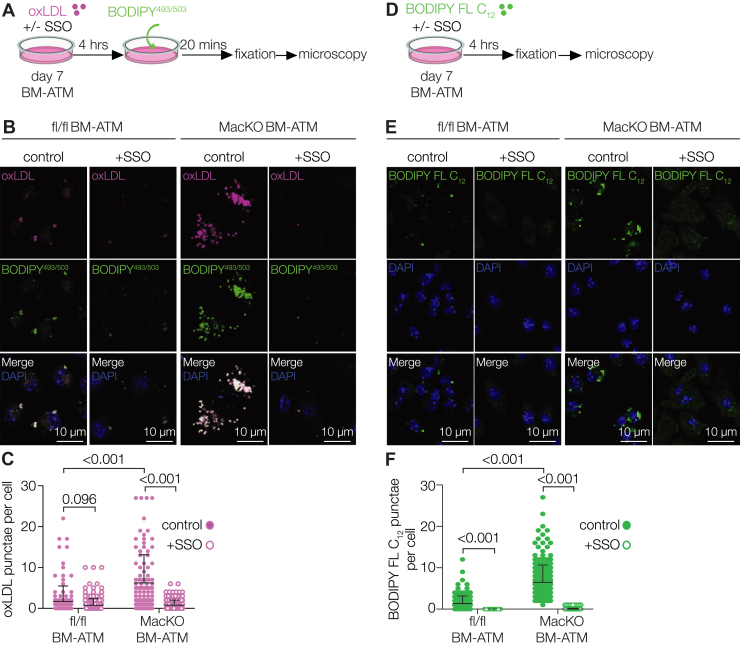
**Siah2 regulates lipid uptake in ATMs dependent on CD36**. *A*, schematic illustrating the oxLDL uptake assay. fl/fl and *Siah2*^MacKO^ BM-ATMs were treated with 10 μg/ml Dil-oxLDL in the presence or the absence of 100 μM SSO, followed by BODIPY^493/503^ staining. *B*, confocal images of fl/fl and *Siah2*^MacKO^ BM-ATMs in the presence or the absence of SSO showing oxLDL (*magenta*) uptake and its colocalization with LDs (BODIPY^493/503^, *green*). *Red signals* were converted to *magenta* using the LUT function in ImageJ. *C*, quantification of oxLDL number per cell shown in *B*. *D*, schematic illustrating BODIPY FL C_12_ uptake assay. fl/fl and *Siah2*^MacKO^ BM-ATMs were treated with 6 μM BODIPY FL C_12_ in the presence or the absence of 100 μM SSO. *E* and *F*, confocal images (*E*) and quantification (*F*) of BODIPY FL C_12_ (*green*) uptake in fl/fl and *Siah2*^MacKO^ BM-ATMs in the presence or the absence of SSO. The scale bars represent 10 μm. Statistics are reported as mean ± SD using two-way ANOVA with Tukey’s multiple comparisons test. *n* = 3 male mice per group. *p* Values are indicated on the graphs. ATM, adipose tissue macrophage; BM-ATM, bone marrow–derived ATM; FL, full length; fl/fl, *Siah2*^flox/flox^; LD, lipid droplet; MacKO, *Siah2*^MacKO^; oxLDL, oxidized low-density lipoprotein; Siah2, seven in absentia homolog 2; SSO, sulfosuccinimidyl oleate.

CD36 is associated with increased oxidized low-density lipoprotein (oxLDL) uptake in macrophages (42, 44), leading to foam cell formation with a proinflammatory phenotype (45, 46). Because we found that Siah2 loss in BM-ATMs increases CD36 protein expression (Fig. 4, *B* and *C*) and elevates inflammation (Fig. 2), we hypothesized that these phenotypes could be linked to oxLDL uptake. To test this, we incubated BM-ATMs with Dil-labeled oxLDL followed by BODIPY^493–503^ staining (Fig. 5*A*). Indeed, oxLDL uptake was significantly increased in *Siah2*^MacKO^ BM-ATMs compared with fl/fl BM-ATMs under control conditions (Fig. 5, *B* and *C*). Interestingly, in both fl/fl and *Siah2*^MacKO^ BM-ATMs, oxLDL colocalized with LDs (Fig. 5*B*), suggesting that oxLDL, after being taken up, was trafficked to the LDs. Because CD36 upregulation is heavily implicated in oxLDL uptake, we next assessed if elevated oxLDL uptake in *Siah2*^MacKO^ BM-ATMs was dependent on CD36. To test this, we incubated the BM-ATMs with Dil-labeled oxLDL for 4 h in the presence of sulfosuccinimidyl oleate (SSO), a pharmacological inhibitor of CD36 (Fig. 5*A*). We found that CD36 inhibition in fl/fl BM-ATMs only moderately decreased oxLDL uptake, whereas in *Siah2*^MacKO^ BM-ATMs, oxLDL uptake was markedly prevented by CD36 inhibition (Fig. 5, *B* and *C*). This indicates that macrophage-specific Siah2 deficiency in BM-ATMs induces oxLDL uptake in a CD36-dependent manner.

As a fatty acid transporter, CD36 is also crucial for facilitating the transport of long-chain fatty acids in different cell types, including adipocytes and immune cells (38). Notably, the BODIPY FL C_12_ fatty acid tracer is used to understand the mechanisms underlying long-chain fatty acid uptake, as BODIPY FL C_12_ constitutes an overall chain length that is equivalent to that of an 18-carbon fatty acid (47). Thus, we used BODIPY FL C_12_ to test if macrophage-specific Siah2 depletion induces long-chain fatty acid uptake in BM-ATMs, dependent on CD36. For this, we incubated BM-ATMs with BODIPY FL C_12_ for 4 h in the presence or the absence of SSO and visualized BODIPY FL C_12_ uptake (Fig. 5*D*). Under control conditions, Siah2 loss in BM-ATMs led to increased BODIPY FL C_12_ uptake, which was abolished by CD36 inhibition (Fig. 5, *E* and *F*). Taken together, our results indicate that macrophage-specific Siah2 deficiency in BM-ATMs leads to increased uptake of oxLDL and long-chain fatty acids dependent on CD36, contributing to elevated lipid accumulation.

### Lipid exposure in ATMs upregulates lysosomal enzymes and induces lipolysis in a Siah2-independent manner

**Figure 6:**
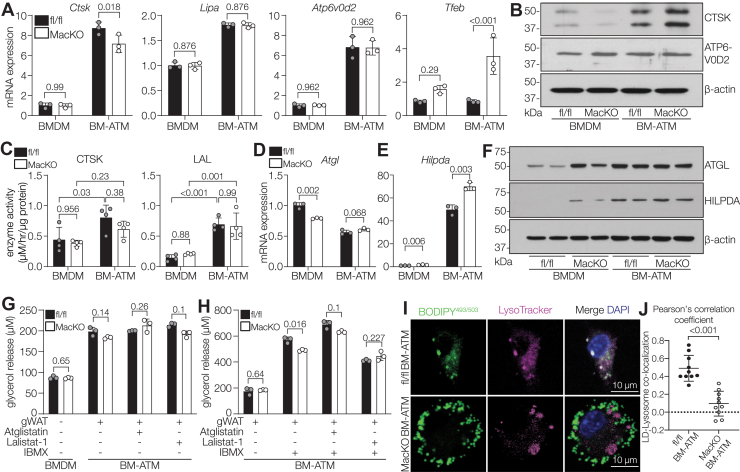
**Lysosomal program is upregulated in ATMs independent of Siah2**. *A*, relative mRNA expression of lysosomal genes in fl/fl and *Siah2*^MacKO^ BMDMs and BM-ATMs. *B*, representative Western blot of lysosomal enzymes relative to β-actin in fl/fl and *Siah2*^MacKO^ BMDMs and BM-ATMs. The β-actin panel is the same as used in Figure 3*F*. The blot shown in Figure 3*F* was reprobed for the CTSK image in *B*. *C*, enzyme activity of CTSK and LAL in fl/fl and *Siah2*^MacKO^ BMDMs and BM-ATMs. *D* and *E*, relative mRNA expression of *Atgl* (*D*) and *Hilpda* (*E*) in fl/fl and *Siah2*^MacKO^ BMDMs and BM-ATMs. *F*, representative Western blot of ATGL and HILPDA relative to β-actin in fl/fl and *Siah2*^MacKO^ BMDMs and BM-ATMs. *G*, basal glycerol release in fl/fl and *Siah2*^MacKO^ BMDMs and BM-ATMs treated with vehicle or 50 μM Atglistatin or 0.1 μM Lalistat-1 for 24 h. *H*, glycerol release in fl/fl and *Siah2*^MacKO^ BM-ATMs treated with vehicle or 50 μM Atglistatin or 0.1 μM Lalistat-1 for 24 h with or without 0.5 mM IBMX (activated lipolysis). *I* and *J*, representative confocal images (*I*) and quantification (*J*) of fl/fl and *Siah2*^MacKO^ BM-ATMs stained for BODIPY^493/503^ (LD, *green*), LysoTracker (lysosomes, *magenta*), and DAPI (nuclei, *blue*) showing colocalization of LD and lysosomes. The *scale bar* represents 10 μm. *Red* signals of LysoTracker were converted to *magenta* using the LUT function in ImageJ. Statistics are reported as mean ± SD using an unpaired *t* test with Welch’s correction, and each dot denotes technical replicates representative of three to five independent experiments. *A*, *D*, *E*, *G*, *H*, and *J*, statistics are reported as mean ± SD using two-way ANOVA with Tukey’s multiple comparisons test (*C*). *p* Values are indicated on the graphs. ATGL, adipose triglyceride lipase; ATM, adipose tissue macrophage; BM-ATM, bone marrow–derived ATM; BMDM, bone marrow–derived macrophage; CTSK, cathepsin K; DAPI, 4′,6-diamidino-2-phenylindole; fl/fl, *Siah2*^flox/flox^; HILPDA, hypoxia-inducible LD-associated protein; IBMX, 3-isobutyl-1-methylxanthine; LAL, lysosomal acid lipase; LD, lipid droplet; MacKO, *Siah2*^MacKO^; Siah2, seven in absentia homolog 2.

Previous reports demonstrated that fatty acid and lipoprotein uptake leads to lipolysis in macrophages (48, 49). Thus, we asked whether macrophage-specific Siah2 depletion alters lipolysis in our model. Lipolysis occurs *via* two major pathways—LD-specific autophagy or lipophagy mediated by lysosomal acid lipase (LAL) (48, 50) or nonlysosomal lipolysis, mediated by adipose triglyceride lipase (ATGL) and hormone-sensitive lipase (51). First, we analyzed the gene expression of lysosomal biogenesis factor *Tfeb* (Transcription Factor EB); lysosomal enzymes, *Ctsk* (cathepsin K), *Lipa* (lysosomal lipase), and *Atp6v0d2* (lysosomal v-ATPase); and found no significant difference between fl/fl BM-ATMs and *Siah2*^MacKO^ BM-ATMs (Fig. 6*A*). Interestingly, mRNA expression of the lysosomal enzymes was markedly upregulated with lipid challenge in the BM-ATMs compared with BMDMs, regardless of genotype (Fig. 6*A*). This increased gene expression of lysosomal enzymes in the BM-ATMs translated into increased CTSK protein expression and increased lysosomal enzyme activity in the BM-ATMs compared with BMDMs (Fig. 6, *B* and *C*). These results agree with a previous study that showed obesity leads to enhanced lysosomal gene expression in the ATMs (21). Although CTSK protein level slightly increased in the *Siah2*^MacKO^ BM-ATMs compared with fl/fl BM-ATMs, we observed no changes in the ATP6V0D2 protein expression across genotypes or conditions (Fig. 6*B*), suggesting that Siah2 depletion in BM-ATMs does not interfere with lysosomal acidification. Together, our data indicate that Siah2 does not regulate the lipid-activated lysosomal program in ATMs and suggest that impaired lysosomal enzyme activity is not a contributing factor to the excess lipid accumulation in Siah2-deficient BM-ATMs.

Next, we asked whether macrophage-specific Siah2 depletion impacts autophagy in ATMs. We observed no significant changes in the mRNA expression of major autophagy genes, including *p62*, *Becn1*, *Lc3b*, *Atg3*, and *Atg14*, between fl/fl BM-ATMs and *Siah2*^MacKO^ BM-ATMs (Fig. S4*A* and Table S1). p62 is the autophagy receptor that binds to ubiquitylated proteins or organelles targeted for degradation (52). Correlated with the autophagy gene expression result (Fig. S4*A*), the p62 protein expression between fl/fl and *Siah2*^MacKO^ remained unaltered in the BMDMs and BM-ATMs, indicating that autophagic flux is maintained even with lipid exposure in the macrophages irrespective of genotypes (Fig. S4, *B* and *C*). Furthermore, we found that macrophage-specific Siah2 loss in BMDM and BM-ATMs increases the expression of autophagosomal marker LC3B (Fig. S4, *B* and *C*), which is indicative of autophagy activation. Altogether, these data show that macrophage-specific Siah2 depletion does not impair lysosomal function and may upregulate autophagic responses during lipid challenges in the ATMs.

We next analyzed the nonlysosomal lipolysis. The gene expression of *Atgl*, the rate-limiting enzyme in the cytosolic lipolysis pathway, diminished in response to Siah2 loss in BMDMs and further decreased in the BM-ATMs in a genotype-independent manner (Fig. 6*D*). Notably, the gene expression of ATGL inhibitors, hypoxia-inducible LD-associated protein (*Hilpda*) (53) and G0/G1 switch gene 2 (*G0s2*) (51), was markedly upregulated in Siah2-depleted macrophages (Figs. 6*E* and S4*D*). Thus, we hypothesized that Siah2 deficiency may downregulate ATGL protein expression by elevating the expression of ATGL inhibitor HILPDA in BM-ATMs. We found that ATGL and HILPDA protein expression increased in the BMDMs in response to macrophage-specific Siah2 loss and further increased by the addition of gWAT (Fig. 6*F*). Notably, we observed a slight increase in the HILPDA protein expression in the Siah2-depleted BM-ATMs compared with fl/fl BM-ATMs (Fig. 6*F*). These results suggest that Siah2 modulates ATGL and HILPDA expression levels in the BMDMs, but lipid exposure is the over-riding influence on lipolytic protein expression in the BM-ATMs.

The HILPDA protein is typically observed at the 10 kDa molecular weight mark in an immunoblot (53). In our experience, we observed HILPDA at a higher molecular weight (∼60 kDa) (Fig. 6*F*). To exclude the possibility that the HILPDA expression at ∼60 kDa is a nonspecific band, we treated the HILPDA antibody with the HILPDA neutralizing peptide prior to immunoblotting. We show that in our coculture model, HILPDA expression was blocked by the HILPDA neutralizing peptide (Fig. S4*E*), confirming the specificity of the HILPDA antibody we used in our study.

Finally, we evaluated whether macrophage-specific Siah2 loss affects lipolysis when the BMDMs are exposed to gWAT. We assayed glycerol release as a measure of lipolysis under both basal and stimulated conditions. Basal lipolysis was increased in the BM-ATMs compared with BMDMs, with no significant difference between fl/fl and *Siah2*^MacKO^ (Figs. 6*G* and S4*F*). Furthermore, pharmacological inhibition of lipases ATGL and LAL by Atglistatin and Lalistat-1, respectively, did not alter basal glycerol release in the BM-ATMs of either genotype (Figs. 6*G* and S4*F*), indicating that Siah2 does not regulate lipid-mediated basal lipolysis in the BM-ATMs.

Catecholamines are well-known inducers of lipolysis that act by binding to and activating a β-adrenergic receptor, leading to cAMP production and subsequent induction of lipase activity and glycerol release (54). However, macrophages degrade catecholamines in a mechanism driven by upregulated GDF3 (55), a phenotype we observed in Siah2-depleted BM-ATMs (Fig. 2*A*). Thus, we bypassed catecholamine receptor binding to stimulate lipolysis by using the phosphodiesterase inhibitor, 3-isobutyl-1-methylxanthine (IBMX), to increase cAMP and stimulate lipolysis. A 24 h incubation with 0.5 mM IBMX showed a ∼3-fold increase in glycerol release in the BM-ATMs (Figs. 6*H* and S4*G*). Unlike basal conditions, we observed a small, but statistically significant, decrease in stimulated lipolysis in *Siah2*^MacKO^ BM-ATMs compared with fl/fl BM-ATMs (Fig. 6*H*). However, this genotype-dependent decline in lipolysis was not evident in Atglistatin-treated or Lalistat-1-treated BM-ATMs (Fig. 6*G*), suggesting Siah2 regulates stimulated lipolysis in BM-ATMs in an ATGL- or LAL-independent manner. Notably, inhibiting LAL under stimulated conditions decreases glycerol release in BM-ATMs irrespective of genotypes (Figs. 6*H* and S4*G*), indicating that LAL is active in ATMs when lipolysis is stimulated. On the contrary, ATGL inhibition by Atglistatin increased glycerol release in fl/fl BM-ATMs and *Siah2*^MacKO^ BM-ATMs under stimulated conditions (Figs. 6*H* and S4*G*), consistent with a link between ATGL-mediated and lysosomal-dependent lipolysis in the BM-ATMs.

Taken together, our results confirm that lipolysis in ATMs is increased in response to lipid exposure, at least partly by the upregulation of lysosomal lipolytic enzymes in a Siah2-independent manner.

### Siah2 regulates LD delivery to lysosomes in ATMs

The interaction between the LD and lysosomal membrane is crucial for the lipid delivery to the lysosome for subsequent degradation (56, 57). Because the lysosomal enzymes are fully functional in the Siah2-depleted BM-ATMs (Fig. 6*C*), we sought to investigate the LD delivery to lysosomes upstream of lysosomal lipolysis. To test this, we visualized lysosomes and LDs using LysoTracker and BODIPY^493–503^, respectively, in our coculture model of BM-ATMs. We found that LDs colocalize with lysosomes in the fl/fl BM-ATMs, meaning that the LDs are delivered to lysosomes for degradation, whereas in the absence of Siah2, LD delivery to the lysosomes is significantly reduced in the BM-ATMs (Fig. 6, *I* and *J*). Next, we sought to determine the possible mechanism driving the Siah2-mediated LD delivery to lysosomes. Notably, recent reports demonstrated that LDs can be directly transported to lysosomes facilitated by lysosomal small GTPase protein ARL8B (58), or LDs can be targeted by Spartin, the selective autophagy receptor for LDs, to the lysosomes for autophagic degradation (59). Based on these studies, we evaluated ARL8B and Spartin protein expression in fl/fl and *Siah2*^MacKO^ macrophages. In line with our imaging results (Fig. 6, *I* and *J*), ARL8B expression was reduced in the *Siah2*^MacKO^ BM-ATMs compared with the fl/fl BM-ATMs, with a slight increase in the *Siah2*^MacKO^ BMDMs compared with fl/fl BMDMs (Fig. S4*H*). However, Spartin expression did not change across genotypes and conditions (Fig. S4*H*). This result corresponds to our finding that macrophage-specific Siah2 loss does not impair autophagy (Fig. S4, *A*–*C*). Altogether, these data hint at a model of reduced lipid delivery to lysosomes in *Siah2*^MacKO^ ATMs that is associated with reduced ARL8B expression independent of autophagy.

LysoTracker analyses and immunolabeling with lysosome-associated membrane protein-1 revealed that the lysosomes in *Siah2*^MacKO^ BM-ATMs were more abundant (Fig. S4, *I* and *J*) and significantly larger (Fig. S4*J*) compared with the fl/fl BM-ATM lysosomes. This indicates that lysosomal abundance is increased in *Siah2*^MacKO^ BM-ATMs in response to high lipid accumulation, as it is previously reported in the context of obesity (21, 37).

### Relationship between lipid accumulation and a proinflammatory phenotype

**Figure 7:**
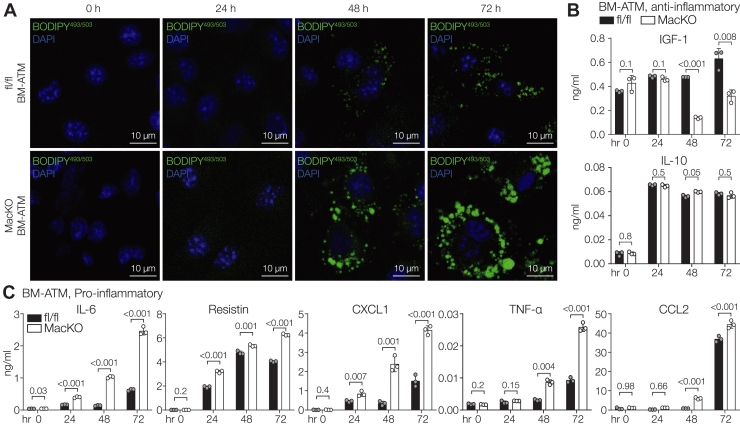
**Lipid accumulation precedes inflammatory responses in *Siah2*^MacKO^ BM-ATMs**. *A*, fl/fl and *Siah2*^MacKO^ BM-ATMs were stained for BODIPY^493/503^ (LD, *green*) and DAPI (nuclei, *blue*) at the indicated timepoints following coculture with gWAT. The scale bar represents 10 μm. *B* and *C*, conditioned media from fl/fl and *Siah2*^MacKO^ BM-ATMs were harvested at the indicated timepoints following coculture with gWAT and subjected to Luminex assay. Concentration (ng/ml) of anti-inflammatory (*B*) and proinflammatory (*C*) factors in the BM-ATM conditioned media. Statistics are reported as mean ± SD using an unpaired *t* test with Welch’s correction. Each dot denotes technical replicates and is representative of two independent experiments. *n* = 2 to 3 male mice per group. *p* Values are indicated on the graphs. BM-ATM, bone marrow–derived adipose tissue macrophage; DAPI, 4′,6-diamidino-2-phenylindole; fl/fl, *Siah2*^flox/flox^; gWAT, 4′,6-diamidino-2-phenylindole; LD, lipid droplet; MacKO, *Siah2*^MacKO^; Siah2, seven in absentia homolog 2.

Next, we asked if lipid accumulation preceded the increased secretion of the proinflammatory we observed in the *Siah2*^MacKO^ BM-ATMs. We assessed neutral lipid accumulation in the fl/fl and *Siah2*^MacKO^ BM-ATMs over the 72 h period after initiating incubation of the BMDMs with AT. Lipid accumulation is apparent at 48 h postincubation with the characteristically higher levels appearing in the *Siah2*^MacKO^ BM-ATMs (Fig. 7*A*). Changes in secretion of insulin-like growth factor 1 as an anti-inflammatory marker decrease in the *Siah2*^MacKO^ BM-ATMs at 48 h, but IL-10 levels are unaffected by lipid accumulation (Fig. 7*B*). Significant differences between the genotypes begin to appear at 24 h for a subset of the proinflammatory markers (IL-6, resistin, and CXCL1), whereas upregulation of other proinflammatory markers (TNFα, CCL2) occurs in the *Siah2*^MacKO^ BM-ATMs at the 48 h mark when lipid accumulation is apparent (Fig. 7*C*). Our results point to a complex relationship between lipid accumulation and a proinflammatory phenotype in the *Siah2*^MacKO^ BM-ATMs, where prolonged lipid accumulation is strongly associated with substantial upregulation of each of the proinflammatory proteins.

### Imbalance between lipid uptake and lipid degradation results in elevated lipid content in *Siah2*^MacKO^ ATMs

**Figure 8:**
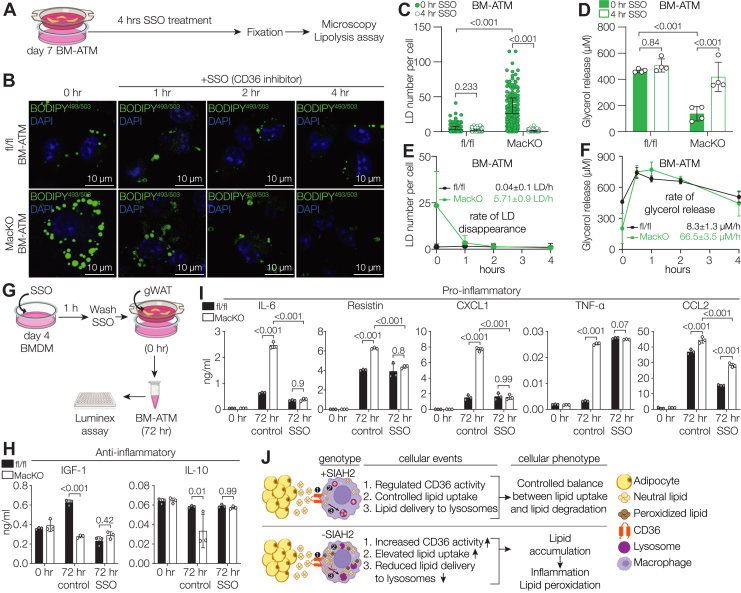
**CD36 inhibition resolves lipid accumulation in Siah2-deficient BM-ATMs**. *A*, *schematic* showing the protocol for SSO treatment followed by microscopy and lipolysis assay. *B*, representative confocal images of fl/fl and *Siah2*^MacKO^ BM-ATMs showing the time course of LD disappearance in the presence or the absence of SSO. *C*, quantification of LD number per cell fl/fl and *Siah2*^MacKO^ BM-ATMs at 0 h and 4 h of SSO treatment, shown in *B*. *D*, glycerol release (μM) at 0 h and 4 h of SSO treatment in fl/fl and *Siah2*^MacKO^ BM-ATMs in the presence of IBMX (0.5 mM). *E*, quantification of LD number per cell at the indicated time points during SSO treatment, shown in *B*. Rate of LD disappearance was determined over 4 h of SSO treatment. *F*, rate of glycerol release (μM) over 4 h of SSO treatment in fl/fl and *Siah2*^MacKO^ BM-ATMs in the presence of IBMX (0.5 mM) at the indicated time points. *G*, *schematic* showing protocol for SSO treatment followed by the Luminex assay. fl/fl and *Siah2*^MacKO^ BMDMs were treated with SSO for 1 h prior to coculture with gWAT. Media were harvested after 3 days of coculture and subjected to the Luminex assay. *H*–*I*, concentration (ng/ml) of anti-inflammatory (*H*) and proinflammatory (*I*) factors in the conditioned media of fl/fl and *Siah2*^MacKO^ BM-ATMs at 0 h and 72 h with or without SSO measured by Luminex assay. *J*, macrophage-specific Siah2 loss in BM-ATMs results in increased lipid uptake dependent on CD36 and reduced lipid delivery to lysosomes, together contributing to excessive lipid accumulation, which results in lipid peroxidation and a proinflammatory phenotype. Statistics are reported as mean ± SD using two-way ANOVA with Tukey’s multiple comparisons test. Each dot denotes technical replicates. *n* = 2 to 3 male mice per group are representative of three independent experiments. *p* Values are indicated on the graphs. BM-ATM, bone marrow–derived adipose tissue macrophage; BMDM, bone marrow–derived macrophage; fl/fl, *Siah2*^flox/flox^; gWAT, gonadal white adipose tissue; IBMX, 3-isobutyl-1-methylxanthine; LD, lipid droplet; MacKO, *Siah2*^MacKO^; Siah2, seven in absentia homolog 2; SSO, sulfosuccinimidyl oleate.

Excessive lipid accumulation occurs in the Siah2-deficient BM-ATMs despite functional lipolytic enzyme activity, suggesting that excessive lipid accumulation and inflammation is caused by elevated lipid uptake and reduced lipid delivery to lysosomes. Thus, we hypothesized that blocking lipid uptake *via* CD36 inhibition would reduce lipid load in the Siah2-depleted BM-ATMs and restore balance between lipid influx and degradation. To test if this is true for *Siah2*^MacKO^ BM-ATMs, we treated the cells with CD36 inhibitor SSO for a short time course of 4 h to measure lipid disappearance and lipolysis (Fig. 8*A*). In fl/fl BM-ATMs, we found no significant changes in LD number per cell after 4 h of CD36 inhibition (Fig. 8, *B*, *C*, *E*), whereas in the *Siah2*^MacKO^ BM-ATMs, CD36 inhibition significantly decreased the number of LDs (Fig. 8, *B*, *C*, *E*). Thus, preventing CD36-mediated lipid uptake in the *Siah2*^MacKO^ BM-ATMs relieves the high lipid load, leading to the clearance of accumulated lipids.

Consistent with the lipid clearance, we observed a significant increase in lipolysis with CD36 inhibition in the *Siah2*^MacKO^ BM-ATMs, whereas no comparable increase in lipolysis was evident in the fl/fl BM-ATMs over 4 h (Fig. 8*D*). Because the amount of lipolytic substrate (LDs) at baseline was significantly more in the *Siah2*^MacKO^ BM-ATMs compared with fl/fl BM-ATMs (Fig. 8, *C* and *E*), the overall rate of glycerol release was substantially higher in the *Siah2*^MacKO^ BM-ATMs during the 4 h time course (Fig. 8*F*). It is interesting to note that, in the *Siah2*^MacKO^ BM-ATMs, a large portion of the accumulated LDs were degraded in the first hour of SSO treatment (Fig. 8, *B* and *E*). Subsequently, the maximum amount of glycerol release in the *Siah2*^MacKO^ BM-ATMs occurred at the 1 h timepoint (Fig. 8*F*), and by the end of 2 h in SSO treatment, the lipid content and glycerol release in *Siah2*^MacKO^ BM-ATMs resembled that in fl/fl BM-ATMs (Fig. 8, *E* and *F*).

To determine if reduced lipid uptake alters secretion of inflammatory markers, fl/fl and *Siah2*^MacKO^ BMDMs were pretreated with SSO as a CD36 inhibitor prior to incubation with AT to form the BM-ATMs over a 72 h time course (Fig. 8*G*). In the absence of CD36-mediated lipid uptake, anti-inflammatory proteins are similarly regulated in the fl/fl and *Siah2*^MacKO^ BM-ATMs (Fig. 8*H*), but a disconnect persists between the proinflammatory proteins (Fig. 8*I*), where TNFα remains elevated in the *Siah2*^MacKO^ BM-ATMs, and CCL2 secretion is reduced in both genotypes but is higher in the *Siah2*^MacKO^ BM-ATMs. In contrast, CXCL1, IL-6, and resistin are not upregulated in the *Siah2*^MacKO^ BM-ATMs when excess lipid uptake and lipid accumulation are inhibited. We also found that lipid accumulation is unaffected by inhibition of *Nos-2* expression in the fl/fl or *Siah2*^MacKO^ BM-ATMs (Fig. S5).

These results strongly indicate that macrophage-specific Siah2 loss in BM-ATMs causes excessive lipid accumulation because of unregulated CD36-mediated lipid uptake that can be resolved by the fully functional lysosomal machinery once lipid uptake is reduced. Our results further indicate that lipid accumulation precedes upregulation of a subset of proinflammatory markers. Thus, these data strengthen our hypothesis that Siah2 acts as a molecular sensor that monitors the balance between lipid influx and clearance in ATMs to impact an inflammatory phenotype in lipid-laden ATMs.

## Discussion

In this study, we used an *ex vivo* model of BM-ATM (21) to explore a role for the Ub ligase Siah2 in ATM phenotypic flexibility in response to the challenge of processing lipids in chronic obesity. We found that macrophage Siah2 limits CD36-mediated lipid uptake and the inflammatory responses associated with excess lipid accumulation in ATMs and that the absence of Siah2 converts lipid-associated macrophages to a foam cell–like inflammatory phenotype (Fig. 8*J*).

Chronic, low-grade inflammation in the AT with infiltrating monocyte–derived macrophages into the AT is considered a driver of obesity-related metabolic dysfunction (4, 5, 6). Although the ATMs promote inflammation, recent evidence also points to a homeostatic role for the ATMs by buffering the lipids released from dying or dysfunctional adipocytes and promoting AT remodeling (7, 60, 61). A growing appreciation of the homeostatic role of ATMs coincides with evidence that, contrary to the earlier, straightforward model of proinflammatory/classically activated (M1) or anti-inflammatory/alternatively activated (M2) macrophages, the ATMs display diverse phenotypes in response to metabolic challenges that are characterized by their transcriptional profile and function (8, 62). As an example, data showing the anti-inflammatory “M2” macrophages are found at crown-like structures (CLSs) surrounding dying adipocytes (61) were followed by identifying another population of ATMs at the CLS that are proinflammatory and lipid laden (8). More recent data characterized a different subset of lipid-laden ATMs at the CLS as “lipid-associated macrophages” or LAMs identified by upregulated genes encoding proteins involved in lysosomal function, lipid uptake, and lipid metabolism (8, 9), with the lipoprotein receptor TREM2 as the signature marker of the LAMs in AT (9). These ATMs share some characteristics with the “metabolically activated” macrophage (MMe) at the CLS that functions to buffer lipid levels in AT when metabolically challenged by obesity (60).

When processing lipids from dying adipocytes, ATMs at the CLS promote AT remodeling by releasing lipids to support the formation of new adipocytes (61). However, as the LAMs become lipid laden with chronic lipid exposure, they undergo a phenotypic switch to a proinflammatory phenotype (7, 8, 9) associated with obesity-related insulin resistance (4, 6). Although the current data support LAM functioning as homeostatic regulators in AT, the mechanisms underlying the dynamic ability of ATMs to switch phenotypes in response to environmental cues or how these mechanisms fail in the lipid-laden ATMs under the metabolic stress of obesity are less well understood. However, it is clear that switching phenotypes in the ATMs depends on responding to the metabolic stress by altering signaling pathways and shifting protein homeostasis in the ATMs (8, 9, 60).

The UPS is the major nonlysosomal protein degradation pathway in macrophages, and its activity is required for macrophages to adapt to changing environmental stimuli (10, 63). The hierarchical enzyme system relies on the Ub ligases to specify protein targets for degradation, and the Ub ligase Siah2 has emerged as a pivotal stress response sensor in hypoxia, immune signaling, and tumor biology (14, 15, 64). In earlier studies, we found that global deletion of Siah2 prevents obesity-related insulin resistance and inflammation (19), although Siah2 promotes adipogenesis by modulating the relative stability of proteins in adipocyte precursors that impact commitment to adipogenesis (18).

*Siah2* mRNA also colocalizes with ATMs at CLS, and *Siah2* is present in CD11b^+^ macrophages isolated from AT stromal cells (18), suggesting a role for Siah2 in ATM biology. However, macrophage-specific deletion of Siah2 does not affect monocyte differentiation into macrophages, indicating the role of Siah2 in adipocyte biology is distinct from that in macrophages. Rather than regulating the conversion of monocytes to macrophages, Siah2 functions in fully differentiated macrophages.

The ability of macrophages to clear dying cells is particularly challenging in obese AT, where dying adipocytes are primarily composed of neutral lipids that require processing in the ATMs. With chronic obesity, a subgroup of lipid-laden ATMs resembling foam cells occurs in human visceral fat (65). These foam cells occur when macrophages fail to maintain lipid homeostasis because of high lipid levels. In atherosclerotic conditions, upregulation of CD36 on macrophages promotes increased uptake of oxLDL, leading to foam cell formation (42, 44, 45). The lipid-laden foamy macrophages are inflammatory (45), enriched with TREM2 (66), and display lipid peroxidation (67). Siah2-depleted ATMs phenotypically resemble foam cells with high levels of lipid accumulation in large LDs because of increased fatty acid and oxLDL uptake associated with increased expression of TREM2 and CD36. Notably, Siah2 deficiency leads to proinflammatory cytokine and chemokine secretion, increased lipid peroxidation, stimulated adipocyte lipolysis, and colocalization of oxLDL with the LDs, all characteristics of foam cells in atherosclerosis (29, 45, 67). This is consistent with sequestering oxidized lipids in LDs to prevent lipotoxicity (68) in the ATMs. On the other hand, Siah2-deficient ATMs have increased lysosomal abundance correlated with elevated lipid accumulation, increased FABP4, and some neutral lipid accumulation unassociated with perilipin-2 enclosed LDs, characteristics observed in ATMs during obesity (21, 37). Thus, Siah2-depleted ATMs exhibit multiple features of lipid-laden foam cells as well as canonical features of LAM ATMs, a phenotype that is distinct from other lipid-laden ATMs.

Although the Siah2-deficient macrophages have foam cell–like characteristics, lysosomal function is unimpaired. Rather than acting simply as an endpoint for degrading cellular contents, lysosomes are integral to regulating lipid metabolism and maintaining homeostasis in macrophages (48, 50). In atherosclerotic foam cells, lipid accumulation is associated with lysosomal dysfunction, and there is evidence that induction of lysosomal biogenesis coupled with autophagy may reverse proinflammatory lipid accumulation in the atherosclerotic foam cells (69). Lysosomal markers are upregulated in ATMs with obesity (21), and our results indicate that enhanced expression and activity of select lysosomal enzymes in response to excess lipid exposure in ATMs are mechanistically independent of Siah2. However, despite the fully functional autophagy, lysosomal enzyme activity, and lysosomal biogenesis in the Siah2-deficient ATMs, compromised lipid clearance from LDs corresponded with impaired LD localization with the lysosomes and stunted lipid delivery to the lysosome. Thus, autophagy is not the limiting factor in delivering lipid cargos to the functional lysosomes in the Siah2-deficient ATMs. However, LAL activity is required for autophagic lipid degradation (50), and we cannot exclude the possibility that some LD contents were degraded in the lysosomes, as inhibition of the LAL reduced lipolysis in the Siah2-depleted ATMs under stimulated conditions.

Although activated cytosolic lipolysis is reduced in the Siah2-deficient ATMs, pharmacological ATGL inhibition did not alter lipolysis under basal or stimulated conditions regardless of the presence of Siah2. However, the hypoxia-inducible LD-associated protein and the G0/G1 switch gene 2 are upregulated in the Siah2-deficient ATMs. Both proteins limit lipolysis by inhibiting ATGL activity (51, 53). While our study does not establish a definitive role for HILPDA or G0S2 in reduced delivery of lipids to the lysosome from the LDs, upregulation of both genes is consistent with a mechanism to sequester reactive lipids to limit lipotoxicity in the Siah2-deficient ATMs.

The indispensable role of LDs in macrophage function is illustrated by close communication of LDs with other organelles to support functional plasticity in macrophages (57). When lipid uptake exceeds degradation, the excess lipid accumulation leads to lipotoxicity, insulin resistance, and metabolic dysfunction (68). In our model, expansion of the number of LDs was related to elevated lipid uptake rather than defects in the release of lipids from the LDs. Inhibiting CD36-mediated fatty acid and oxLDL uptake in the Siah2-deficient ATMs resolved accumulation of excess lipids in the large LDs within an hour, confirming that lipid degradation is intact in the Siah2-deficient ATMs and that the foam cell–like phenotype of the Siah2-deficient ATMs reflects Siah2 acting to modulate lipid uptake in ATMs.

We and others have reported that Siah2 transcriptionally regulates lipid metabolism in different physiological contexts, including circadian regulation of gene expression, AT inflammation, and cancer (70, 71, 72). In addition, we reported evidence that Siah2 selectively regulates PPARγ activity in AT by regulating expression of a subset of PPARγ targets, including CD36 as a canonical PPARγ target gene (16, 19, 20). In macrophages, PPARγ acts as a master regulator of lipid metabolism and an anti-inflammatory mediator (73, 74). However, elevated PPARγ activity and unchecked CD36-mediated lipid uptake in macrophages are proatherogenic and contribute to foam cell lipid accumulation (45). In our model, CD36-mediated lipid uptake does not appear to be balanced by oxLDL efflux, suggesting that Siah2 is acting predominantly on CD36, although expression of other PPARγ1 target proteins, such as FABP4, is also elevated.

Although ligand-dependent PPARγ activation is associated with increased turnover of ligand-bound PPARγ in adipocytes, we found that Siah2 regulates the mRNA and protein levels of select PPARγ1 targets, including CD36, FABP4, and HILPDA in ATMs, without affecting PPARγ1 steady-state levels. This suggests that Siah2 in macrophages regulates PPARγ1 target protein expression independent of ligand-mediated PPARγ1 turnover. This possibility is supported by the presence of Siah2 in the cytoplasm, coupled with Siah2-mediated ubiquitylation of CD36. However, the lack of known Siah2-binding sites in CD36 points to an indirect interaction of Siah2 and CD36. Although we do not identify possible Siah2-interacting cytoplasmic partners, as a RING-type Ub ligase, Siah2 acts as a scaffold to facilitate Ub transfer from an E2-conjugating enzyme to the targeted protein (75), and like other RING-type Ub ligases, Siah2 associates with adaptor proteins to mediate their activities (76). A cytoplasmic function of Siah2 does not rule out Siah2 acting in the nucleus by interacting with the nuclear receptor corepressor protein 1 (NCoR1) to regulate PPARγ transcriptional activity (16). Ligand-bound PPARγ stimulation of CD36 activity is inhibited by NCoR1 (77), but a PPARγ–NCoR1 interaction also acts to transrepress expression of proinflammatory genes in macrophages, exemplified by transrepression of NOS2 in macrophages (78). In an atherosclerosis model of macrophage-specific NCoR1 deletion, loss of NCoR1 increased PPARγ-mediated CD36 expression, elevated inflammatory cytokine levels, and led to a foam cell–like phenotype (77). Our model of Siah2-depleted ATMs phenocopies NCoR1 knockout macrophages with upregulated CD36 levels combined with elevated inflammation, including increased NOS2 expression, and excess lipid accumulation resembling foam cells.

While our study does not establish a clear mechanism of action for the role of Siah2 in macrophages, we identify Siah2 as a molecular sensor that monitors CD36-dependent lipid uptake and alters PPARγ transcriptional activity and target protein levels to limit excess lipid accumulation in ATMs. This places Siah2 as a central factor contributing to homeostatic control of ATM lipid metabolism and provides mechanistic detail about how ATMs balance lipid influx and degradation in a challenging lipid environment.

We acknowledge various limitations of our study. BMDMs represent recruited macrophages and not tissue-resident macrophages. However, previous reports show that BM-ATMs show characteristics of tissue-resident ATMs (21, 37). While the *ex vivo* model we used recapitulates the characteristics of ATMs, we do not address how Siah2 specifically influences tissue-resident ATMs in response to metabolic adaptations. During metabolic challenges, LDs communicate extensively with other cell organelles (57). Further investigation is needed to determine how Siah2 regulates the LD–lysosome communication to facilitate lipid delivery to the lysosomes. Moreover, a subset of accumulated lipids in our ATM model does not colocalize with the LD membrane marker, PLIN2. Thus, additional studies are needed to test the possibility that the Siah2-deficient ATMs internalize neutral lipids in adipocyte-derived exosomes *via* endocytic pathways (37). While the increased lipid uptake in our model is a direct consequence of CD36 upregulation, we currently lack evidence that other lipid scavenger receptors, including CD206, scavenger receptor class A-I, and scavenger receptor class B-I (49), may contribute to this phenotype. Mechanistically, this CD36 upregulation is induced by PPARγ activation independent of changes in PPARγ steady-state protein levels. Further studies are needed to assess whether Siah2 modulates PPARγ activation in macrophages independent of targeting ligand-activated PPARγ for degradation.

## Experimental procedures

### Animal care and maintenance

Macrophage-specific *Siah2* knockout (*Siah2*^MacKO^) mice are generated by using the “floxed” homozygous *Siah2*^flox/flox^ mouse crossed with the homozygous LysM-cre mouse (20). The male and female mice were multihoused within a pathogen-free barrier facility with a 12-h light–dark cycle at 24 °C. The mice were fed a normal chow diet (LabDiet 5001) and euthanized at 10 to 12 weeks of age immediately after CO_2_ asphyxiation for isolating bone marrow and/or gWAT tissue. All procedures were approved by the Pennington Biomedical Research Center Animal Care and Use Committee (protocol #PB-22-013).

### *Ex vivo* bone marrow cell differentiation and coculture

Bone marrow cells (BMCs) were obtained and processed from the femur and tibia of male and female mice as described (79). The BMCs were then isolated and cultured in growth media containing Dulbecco's modified Eagle's medium (DMEM)/F12, 10% fetal bovine serum, and 1% penicillin–streptomycin for 24 h. The next day, nonadherent BMCs were counted and plated at 2 × 10^6^ cells per well into 6-well tissue culture plates (Corning Incorporated) or 0.5 × 10^6^ cells per well into 24-well tissue culture plates (Corning Incorporated). BMCs were cultured in differentiation media containing DMEM/F12, 10% fetal bovine serum, 1% penicillin/streptomycin, and 30 ng/ml macrophage-colony stimulating factor (Peprotech, catalog no.: 315-02) for 4 days and differentiated into BMDMs. On day 4, BMDMs were cocultured with gWAT (0.1 g/well; 6-well format) collected from wildtype lean male mice 10 to 12 weeks of age, as described (21). BMDMs cultured in media without gWAT were considered controls. BMDMs were further differentiated into BM-ATM in the presence of gWAT for an additional 3 days. On day 7, BMDMs and BM-ATMs were harvested for Western blot analyses, gene expression analyses, biochemical assays, and immunostaining.

### Reverse transcription–PCR

Cells were harvested in TriReagent (Molecular Research), and total RNA was purified using the RNeasy kit (Qiagen, catalog no.: 74106). RNA (500 ng) was reverse-transcribed using High-Capacity cDNA Reverse Transcription kit (Applied Biosystems, catalog no.: 4368814), and real-Time PCR was performed with SYBR Green (Applied Biosystems, catalog no.: A25742) as described (16). The results were normalized to *18s* mRNA levels and analyzed by the 2^-ΔΔCT^ method using *Siah2*^*f*lox/flox^ BMDM as the calibrator. The list of primers for each gene of interest is provided in Table S1.

### Western blotting

Cells were harvested and sonicated in a nondenaturing buffer containing 50 mM Tris–Cl (pH 7.4), 150 mM NaCl, 1% NP-40 (Igepal), 0.5% sodium deoxycholate, 0.1% SDS with phosphatase inhibitors (2 mM NaVO_4_, 2 mM β-glycerophosphate, and 1 mM NaFl), and protease inhibitors (1 mM PMSF, 1 μM pepstatin, 2.5 μg/ml aprotinin, 4.7 μg/ml leupeptin, 1 μM E-64, 20 μM caspase-1 inhibitor, 20 μM caspase-3 inhibitor, and 20 μM pan-Caspase inhibitor), proteasomal inhibitor (10 μM MG132) and 10 mM *N*-ethylmaleimide. Total protein concentration was measured using the BCA assay kit (Thermo Fisher/Pierce), and 20 μg protein was run in 10% SDS-polyacrylamide (National Diagnostics) gels and subjected to immunoblotting. Nitrocellulose membranes (Bio-Rad, 0.2 μm) were incubated with primary antibodies for 1 to 2 h at room temperature or overnight at 4°C, followed by 1 h incubation with secondary antibody at room temperature. To confirm the specificity of the HILPDA antibody, the HILPDA antibody and HILPDA neutralizing peptide were mixed at a 1:5 ratio in 1x PBS and incubated overnight at 4 °C. The next day, the nitrocellulose membrane was incubated with the neutralization mix for 2 h at room temperature, followed by 1 h incubation with the secondary antibody at room temperature. β-actin was used as a loading control. Band intensities were analyzed using ImageJ software (National Institutes of Health). The list of antibodies is provided in Table S2.

### Immunoprecipitation

Cells were harvested in nondenaturing lysis buffer, and the protein content of the whole-cell extracts was analyzed as described above. Protein extracts (100 μg) were precleared with protein A/G magnetic beads (Pierce, catalog no.: 88802) for 1 h at 4 °C. The resulting supernatant was then incubated with 1 μg of the polyclonal anti-CD36 antibody or polyclonal anti-FABP4 for 1 h at 4 °C. Protein A/G magnetic beads were added to the mixture, and the sample was rotated for an additional hour at 4 ^o^C. Bound CD36, FABP4, and any associated proteins were magnetically pelleted and rinsed with lysis buffer twice. During each rinse, the supernatant was collected as “flow-through.” The bound proteins were then eluted from the protein A/G by incubation at 100 °C for 10 min after the addition of sample buffer containing β-mercaptoethanol. These samples were separated by SDS-PAGE and analyzed by Western blotting with either anti-CD36, anti-FABP4, anti-Siah2, or anti-Ub antibodies.

### Cellular fractionation assay

BM-ATMs were cultured and differentiated on 6-well tissue culture plates. Cells were pretreated with 20 nM leptomycin B (MilliporeSigma, catalog no.: L2913) for 30 min at 37 °C to block nuclear export. The cells were then harvested in hypotonic buffer (10 mM Tris–Cl, pH 7.9 with 1.5 mM MgCl_2_, and 10 mM KCl) with phosphatase and protease inhibitors, along with 1 mM epoxomicin (Boston Biochem) and 10 mM *N*-ethylmaleimide. The harvested cells were incubated on ice for 15 min prior to isolating nuclei using a glass type B Dounce homogenizer (10–12 strokes). Cell lysis was confirmed using Trypan Blue prior to isolating the nuclei by centrifuging at 4 °C for 10 min at 500*g*. The resulting supernatant was retained as the cytoplasmic fraction. The pelleted nuclei were resuspended in nuclear extract buffer (10 mM Tris–Cl, pH 7.4 with 10 mM NaCl, 10 mM MgCl_2_, 0.1% Tween-20, 0.1% Igepal, 0.01% digitonin, and 1% bovine serum albumin) and sonicated. Western blot analysis was carried out on the cytoplasmic and nuclear extract (25 μg each) using anti-Siah2 (1:1000 dilution), anti-Histone 2AX (1:500 dilution), and β-actin (1:1000 dilution).

### CTSK activity assay

CTSK activity in BMDMs and BM-ATMs was measured using the Cathepsin K assay kit (Novus Biologicals, catalog no.: NBP2-54842) according to the manufacturer’s protocol. Briefly, cells were harvested in CTSK cell lysis buffer and incubated for 60 min at 4 °C for complete lysis. Cells were then centrifuged at 16,000*g* for 5 min at 4 °C. The resulting supernatant was incubated with 200 μM Ac-LR–7-amino-4-trifluoromethylcoumarin substrate at 37 °C for 2 h. Fluorescence was measured using a fluorometer equipped with excitation/emission = 400 nm/505 nm filters. CTSK activity was determined by generating a standard curve using free 7-amino-4-trifluoromethylcoumarin (Abcam, catalog no.: ab145587). The results were normalized to total protein and reported as micromolar per hour per microgram of protein.

### LAL activity assay

BMDMs and BM-ATMs were harvested in LAL assay buffer containing 100 mM sodium acetate (pH 4.0) and 1% Triton X-100. Cells were sonicated and centrifuged at 10,000*g* for 15 min at 4 °C. The resulting supernatant was incubated with 200 μM of substrate 4-methylumbelliferyl oleate (MilliporeSigma, catalog no.: 75164) at 37 °C for 30 min. For the negative control, 0.1 μM Lalistat-1 (TOCRIS, catalog no.: 6098) was used. Fluorescence was measured using a fluorometer equipped with excitation/emission = 320 nm/460 nm filters. LAL activity was determined by generating a standard curve using 4-methylumbelliferone (MilliporeSigma, M1381). The results were normalized to total protein and reported as micromolar per hour per microgram of protein.

### Macrophage lipolysis assay

BMDMs and BM-ATMs were cultured and differentiated on 6-well tissue culture plates as described in “*Ex vivo* bone marrow cell differentiation and coculture,” except DMEM/F12 media were replaced by DMEM phenol-free media supplemented with 2 mM l-glutamine (MP Biochemicals, catalog no.: 1680149). Media were removed, and the cells were rinsed with PBS three times. The cells were then incubated with vehicle alone, 50 μM Atglistatin (Cayman Chemical, catalog no.: 15284), or 0.1 μM Lalistat-1 (TOCRIS, catalog no.: 6098), each with or without 0.5 mM IBMX (MilliporeSigma, catalog no.: I5879) for 24 h. For the time-course experiment with CD36 inhibition, cells were pretreated with or without 100 μM SSO (Cayman Chemical, catalog no.: 11211) for a time course of 0 to 4 h. Cells were then washed with 1x PBS and incubated with 0.5 mM IBMX for 24 h. Media were harvested, and glycerol release was determined using the lipolysis assay kit (Abcam, catalog no.: ab185433) according to the manufacturer’s instructions. Rate of glycerol release was determined by the equation ([Gly^t0^ - Gly^t4^]/t; where Gly = glycerol concentration (μm) and t = 4].

### *Ex vivo* AT lipolysis assay

BM-ATMs were cultured and differentiated on 6-well tissue culture plates as described in “*Ex vivo* bone marrow cell differentiation and coculture,” except that DMEM/F12 media were replaced by DMEM phenol-free media supplemented with 2 mM l-glutamine. After coculture, BM-ATM conditioned media were collected. For the lipolysis assay, AT from lean wildtype mice was freshly isolated, washed in PBS, dissected into 25 mg pieces, and equally distributed into a 6-well plate (0.1 g gWAT/well). The AT explants were then incubated with media (DMEM phenol free, low glucose), media with 0.75 nM TNF-α as a control for lipolysis induction *via* inflammatory signaling (80), fl/fl BM-ATM conditioned media, or *Siah2*^MacKO^ BM-ATM conditioned media for 16 h at 37 °C. Media were harvested, and lipolysis was quantified by measuring free fatty acid and free glycerol release in the media by using a free fatty acid assay kit (Abcam, catalog no.: ab65341) and a lipolysis assay kit (Abcam, catalog no.: ab185433), respectively. The lipolysis assays were performed according to the manufacturer’s instructions.

### Superoxide production assay

Superoxide production was determined by the addition of 100 mM of spin trap 5,5-dimethyl-1-pyrroline-*N*-oxide (DMPO, Enzo Life Sciences) into the cell culture media for the duration of coculture with gWAT. The resulting media were harvested and stored at −80 °C until the time of the assay. DMPO-OH/OOH adducts, which are directly proportional to superoxide production, were detected by EPR (Bruker EMX Plus spectroscope) in a quartz flat cell at room temperature. The resulting four-line spectra (1:2:2:1 quartet pattern) were characterized using the hyperfine splitting constant a_N_ = a_H_ = 14.9 G, representative of a DMPO-OH/OOH adduct. Instrument parameters were 20 mW microwave power, 1.0 G modulation amplitude, 1 × 10^5^ gain, 0.163 s time constant, and 80 G scan width with center field 3485 G. To improve the signal-to-noise ratio, spectra were accumulated 3x for each sample. Quantitation was carried out by measuring and comparing the amplitudes of the first peaks on each spectrum using the Bruker WinEPR Software and expressed as EPR signal intensity in arbitrary units.

### Luminex assay

BMDMs and BM-ATMs were cultured and differentiated for 7 days (as described above). Media alone and media collected after incubation with gWAT only in the insert were used as negative controls. For CD36 inhibition, day 4 BMDMs were treated with 100 μM SSO for 1 h. Cells were then washed with PBS, and gWAT explants were added for coculture. After 3 days of coculture, media were pooled from three wells (6-well format) per mouse and centrifuged at 16,000*g* for 5 min immediately prior to the assay. The resulting supernatant was processed with the Mouse Luminex Discovery Assay kit (R&D Systems, catalog no.: LXSAMSM) according to the manufacturer’s instructions. All samples and standard dilutions were tested in triplicate, and the mean fluorescence intensity (MFI) was measured using a Bio-Rad Bio-Plex analyzer. The MFI of negative controls was subtracted from the MFI values of each cytokine. A standard curve was then generated for each cytokine to calculate the final concentration in nanograms per milliliter.

### Lipid uptake assay

For all imaging experiments (unless otherwise noted), BMDMs and BM-ATMs were cultured and differentiated on poly-d-lysine (Gibco, catalog no.: A3890401)-coated coverslips (diameter of 12 mm) placed in each well of a 24-well tissue culture plate. After 7 days of differentiation, hanging well inserts containing the gWAT were removed, and 10 μg/ml Dil-oxLDL (Thermo Scientific, catalog no.: L34358) or 6 μM BODIPY FL-C12 fatty acid tracer (Invitrogen, catalog no.: D-3822), each with and without 100 μM SSO, were added to the existing media. The cells were incubated with lipid tracers for 4 h at 37 °C. Cells were washed with PBS three times, followed by fixation for 10 min in 4% paraformaldehyde (PFA; Thermo Scientific) at room temperature. The 4% PFA was removed, and the coverslips were washed with 30 mM glycine in PBS three times. Cells were then mounted with Prolong Gold Antifade Reagent with 4′,6-diamidino-2-phenylindole (DAPI) (Invitrogen, catalog no.: P36931) for microscopy.

### Immunostaining

After 7 days of differentiation, hanging well inserts containing the gWAT were removed, and cells were rinsed with PBS three times. Cells were then fixed in 4% PFA for 10 min, permeabilized in PBS/0.1% Tween-20 for 20 min, and blocked in PBS/1% bovine serum albumin/0.01% Tween-20 for 1 h. Cells were incubated overnight at 4 °C with the following primary antibodies: PLIN2 (Proteintech, catalog no.: 15294-1-AP), F4-80 (Abcam, catalog no.: ab6640), lysosome-associated membrane protein-1 (Invitrogen, catalog no.: 14-1071-82), and Siah2 (Proteintech, catalog no.: 12651-1-AP). Cells were then incubated for 1 h at room temperature with the following secondary antibodies: Goat anti-rabbit AF-488 (Molecular Probes, catalog no.: A11034), Goat anti-rabbit AF-568 (Invitrogen, catalog no.: A11036), and Goat anti-rat AF-647 (Abcam, catalog no.: ab150167). All washing steps were performed with PBS three times. Cells were mounted with Prolong Gold Antifade Reagent with DAPI for microscopy.

### BODIPY and LysoTracker staining

After 7 days of differentiation, hanging well inserts containing the gWAT were removed, and cells were rinsed with PBS three times. Cells were incubated for 15 min with 5 μM of BODIPY^493/503^ (Invitrogen, catalog no.: D-3922) to visualize neutral lipids or 10 μM of BODIPY^581/591^ (Invitrogen, catalog no.: D-3861) to visualize peroxidized lipids at 37 °C. Cells were washed with PBS three times, followed by fixation for 10 min in 4% PFA at room temperature. The 4% PFA was removed, and the coverslips were washed with 30 mM glycine in PBS three times. For identification of acidic lysosomes, cells were incubated for 15 min with 5 μM LysoTracker Deep Red (Invitrogen, catalog no.: L-12492) at 37 °C and washed with PBS three times. Cells were mounted with Prolong Gold Antifade Reagent with DAPI for microscopy.

### Inducible nitric oxide synthase inhibition assay

BMDMs were cultured and differentiated for 4 days (as described above). BMDMs were treated with 100 μM 1400-W (inducible nitric oxide synthase inhibitor, Sigma–Aldrich, catalog no.: 100050-5MG) for 1 h. Cells were then washed with PBS, and gWAT explants were added for coculture. After 3 days of coculture, cells were harvested for RT–PCR. For LD detection, cells were stained with 5 μM BODIPY^493/503^ prior to microscopy, as described above.

### Microscopy and image analysis

A Leica Confocal SP5 microscope equipped with an inverted DMI6000CS camera was used for imaging. Images were acquired with differential interference contrast and sequential imaging with excitation and emission filters of 358/461 (*blue*), 493/504 (*green*), 579/603 *(red*), and 647/667 (*far-red*). All images were analyzed using ImageJ software. Red and far-red colors were converted to magenta using the LUT function in ImageJ. For LD–lysosome colocalization analyses, the Coloc2 function under the “Analyze” tab was used, and Pearson’s correlation coefficient was plotted to generate graphs.

### Statistics

Results are presented as the mean and standard deviation of data from at least three biological replicates. An unpaired *t* test was used to determine the comparison between two groups. Two-way ANOVA was used to determine the comparison between more than two groups. Differences with a *p* value of <0.01 were considered as statistically significant. Statistical analyses were performed, and graphs were generated using GraphPad Prism 10.0 (GraphPad Software).

## Data availability

All available data are presented in the article. Other information underlying this study is available from the corresponding author upon reasonable request.

## Supporting information

This article contains supporting information.

## Conflict of interest

The authors declare that they have no conflicts of interest with the contents of this article.

## References

[1] Jin X., Qiu T., Li L., Yu R., Chen X., Li C. (2023). Pathophysiology of obesity and its associated diseases. Acta Pharm. Sin B.

[2] Emerging Risk Factors C., Wormser D., Kaptoge S., Di Angelantonio E., Wood A.M., Pennells L. (2011). Separate and combined associations of body-mass index and abdominal adiposity with cardiovascular disease: collaborative analysis of 58 prospective studies. Lancet.

[3] Onyemaechi N.O., Anyanwu G.E., Obikili E.N., Onwuasoigwe O., Nwankwo O.E. (2016). Impact of overweight and obesity on the musculoskeletal system using lumbosacral angles. Patient Prefer Adherence.

[4] Xu H., Barnes G.T., Yang Q., Tan G., Yang D., Chou C.J. (2003). Chronic inflammation in fat plays a crucial role in the development of obesity-related insulin resistance. J. Clin. Invest..

[5] Weisberg S.P., McCann D., Desai M., Rosenbaum M., Leibel R.L., Ferrante A.W. (2003). Obesity is associated with macrophage accumulation in adipose tissue. J. Clin. Invest..

[6] Lumeng C.N., DelProposto J.B., Westcott D.J., Saltiel A.R. (2008). Phenotypic switching of adipose tissue macrophages with obesity is generated by spatiotemporal differences in macrophage subtypes. Diabetes.

[7] Kratz M., Coats B.R., Hisert K.B., Hagman D., Mutskov V., Peris E. (2014). Metabolic dysfunction drives a mechanistically distinct proinflammatory phenotype in adipose tissue macrophages. Cell Metab..

[8] Hill D.A., Lim H.W., Kim Y.H., Ho W.Y., Foong Y.H., Nelson V.L. (2018). Distinct macrophage populations direct inflammatory versus physiological changes in adipose tissue. Proc. Natl. Acad. Sci. U. S. A..

[9] Jaitin D.A., Adlung L., Thaiss C.A., Weiner A., Li B., Descamps H. (2019). Lipid-associated macrophages control metabolic homeostasis in a Trem2-Dependent manner. Cell.

[10] Loix M., Zelcer N., Bogie J.F.J., Hendriks J.J.A. (2024). The ubiquitous role of ubiquitination in lipid metabolism. Trends Cell Biol..

[11] Zou Y., Zhang Y., Li M., Cao K., Song C., Zhang Z. (2024). Regulation of lipid metabolism by E3 ubiquitin ligases in lipid-associated metabolic diseases. Int. J. Biol. Macromol.

[12] Govatati S., Kumar R., Boro M., Traylor J.G., Orr A.W., Lusis A.J. (2024). TRIM13 reduces cholesterol efflux and increases oxidized LDL uptake leading to foam cell formation and atherosclerosis. J. Biol. Chem..

[13] Yang S., Wang B., Humphries F., Hogan A.E., O'Shea D., Moynagh P.N. (2014). The E3 ubiquitin ligase Pellino3 protects against obesity-induced inflammation and insulin resistance. Immunity.

[14] Nakayama K., Frew I.J., Hagensen M., Skals M., Habelhah H., Bhoumik A. (2004). Siah2 regulates stability of prolyl-hydroxylases, controls HIF1alpha abundance, and modulates physiological responses to hypoxia. Cell.

[15] Habelhah H., Frew I.J., Laine A., Janes P.W., Relaix F., Sassoon D. (2002). Stress-induced decrease in TRAF2 stability is mediated by Siah2. EMBO J..

[16] Kilroy G., Kirk-Ballard H., Carter L.E., Floyd Z.E. (2012). The ubiquitin ligase Siah2 regulates PPARgamma activity in adipocytes. Endocrinology.

[17] Kilroy G., Burk D.H., Floyd Z.E. (2016). Siah2 protein mediates early events in commitment to an adipogenic pathway. J. Biol. Chem..

[18] Dang T.N., Taylor J.L., Kilroy G., Yu Y., Burk D.H., Floyd Z.E. (2021). SIAH2 is expressed in adipocyte precursor cells and interacts with EBF1 and ZFP521 to promote adipogenesis. Obesity (Silver Spring).

[19] Kilroy G., Carter L.E., Newman S., Burk D.H., Manuel J., Moller A. (2015). The ubiquitin ligase Siah2 regulates obesity-induced adipose tissue inflammation. Obesity (Silver Spring).

[20] Dang T.N., Ghosh B., Panta P.R., Taylor J.M., Kilroy G., Beyl R. (2026). Siah2 is a lipid-meditated metabolic sensor in adipose tissue macrophage. J. Lipid Res..

[21] Xu X., Grijalva A., Skowronski A., van Eijk M., Serlie M.J., Ferrante A.W. (2013). Obesity activates a program of lysosomal-dependent lipid metabolism in adipose tissue macrophages independently of classic activation. Cell Metab..

[22] Lumeng C.N., Bodzin J.L., Saltiel A.R. (2007). Obesity induces a phenotypic switch in adipose tissue macrophage polarization. J. Clin. Invest..

[23] Al-Qahtani A.A., Alhamlan F.S., Al-Qahtani A.A. (2024). Pro-inflammatory and anti-inflammatory interleukins in infectious diseases: a comprehensive review. Trop. Med. Infect. Dis..

[24] Sukhanov S., Higashi Y., Shai S.Y., Vaughn C., Mohler J., Li Y. (2007). IGF-1 reduces inflammatory responses, suppresses oxidative stress, and decreases atherosclerosis progression in ApoE-deficient mice. Arterioscler Thromb. Vasc. Biol..

[25] Jang I.H., Carey A., Kruglov V., Nguyen K., Misialek J.R., Cholensky S.H. (2026). GDF3 promotes adipose tissue macrophage-mediated inflammation via altered chromatin accessibility during aging. Nat. Aging.

[26] Ather J.L., Ckless K., Martin R., Foley K.L., Suratt B.T., Boyson J.E. (2011). Serum amyloid A activates the NLRP3 inflammasome and promotes Th17 allergic asthma in mice. J. Immunol..

[27] Steppan C.M., Bailey S.T., Bhat S., Brown E.J., Banerjee R.R., Wright C.M. (2001). The hormone resistin links obesity to diabetes. Nature.

[28] Qatanani M., Szwergold N.R., Greaves D.R., Ahima R.S., Lazar M.A. (2009). Macrophage-derived human resistin exacerbates adipose tissue inflammation and insulin resistance in mice. J. Clin. Invest..

[29] Song J., Farris D., Ariza P., Moorjani S., Varghese M., Blin M. (2023). Age-associated adipose tissue inflammation promotes monocyte chemotaxis and enhances atherosclerosis. Aging Cell.

[30] Zhang H.H., Halbleib M., Ahmad F., Manganiello V.C., Greenberg A.S. (2002). Tumor necrosis factor-alpha stimulates lipolysis in differentiated human adipocytes through activation of extracellular signal-related kinase and elevation of intracellular cAMP. Diabetes.

[31] Gaschler M.M., Stockwell B.R. (2017). Lipid peroxidation in cell death. Biochem. Biophys. Res. Commun..

[32] Cohen G., Riahi Y., Sasson S. (2011). Lipid peroxidation of poly-unsaturated fatty acids in normal and obese adipose tissues. Arch. Physiol. Biochem..

[33] Ayala A., Munoz M.F., Arguelles S. (2014). Lipid peroxidation: production, metabolism, and signaling mechanisms of malondialdehyde and 4-hydroxy-2-nonenal. Oxid Med. Cell Longev.

[34] Andringa K.K., Udoh U.S., Landar A., Bailey S.M. (2014). Proteomic analysis of 4-hydroxynonenal (4-HNE) modified proteins in liver mitochondria from chronic ethanol-fed rats. Redox Biol..

[35] van Herpen N.A., Schrauwen-Hinderling V.B. (2008). Lipid accumulation in non-adipose tissue and lipotoxicity. Physiol. Behav..

[36] Wong A., Chen S., Yang L.K., Kanagasundaram Y., Crasta K. (2018). Lipid accumulation facilitates mitotic slippage-induced adaptation to anti-mitotic drug treatment. Cell Death Discov..

[37] Flaherty S.E., Grijalva A., Xu X., Ables E., Nomani A., Ferrante A.W. (2019). A lipase-independent pathway of lipid release and immune modulation by adipocytes. Science.

[38] Chen Y., Zhang J., Cui W., Silverstein R.L. (2022). CD36, a signaling receptor and fatty acid transporter that regulates immune cell metabolism and fate. J. Exp. Med..

[39] House C.M., Frew I.J., Huang H.L., Wiche G., Traficante N., Nice E. (2003). A binding motif for Siah ubiquitin ligase. Proc. Natl. Acad. Sci. U. S. A..

[40] Habelhah H., Laine A., Erdjument-Bromage H., Tempst P., Gershwin M.E., Bowtell D.D. (2004). Regulation of 2-oxoglutarate (Alpha-ketoglutarate) dehydrogenase stability by the RING finger ubiquitin ligase Siah. J. Biol. Chem..

[41] Yorek M., Jiang X., Liu S., Hao J., Yu J., Avellino A. (2024). FABP4-mediated lipid accumulation and lipolysis in tumor-associated macrophages promote breast cancer metastasis. Elife.

[42] Tontonoz P., Nagy L., Alvarez J.G., Thomazy V.A., Evans R.M. (1998). PPARgamma promotes monocyte/macrophage differentiation and uptake of oxidized LDL. Cell.

[43] Moore K.J., Rosen E.D., Fitzgerald M.L., Randow F., Andersson L.P., Altshuler D. (2001). The role of PPAR-gamma in macrophage differentiation and cholesterol uptake. Nat. Med..

[44] Nicholson A.C., Frieda S., Pearce A., Silverstein R.L. (1995). Oxidized LDL binds to CD36 on human monocyte-derived macrophages and transfected cell lines. Evidence implicating the lipid moiety of the lipoprotein as the binding site. Arterioscler. Thromb. Vasc. Biol..

[45] Rahaman S.O., Lennon D.J., Febbraio M., Podrez E.A., Hazen S.L., Silverstein R.L. (2006). A CD36-dependent signaling cascade is necessary for macrophage foam cell formation. Cell Metab..

[46] Janabi M., Yamashita S., Hirano K., Sakai N., Hiraoka H., Matsumoto K. (2000). Oxidized LDL-induced NF-kappa B activation and subsequent expression of proinflammatory genes are defective in monocyte-derived macrophages from CD36-deficient patients. Arterioscler. Thromb. Vasc. Biol..

[47] Rambold A.S., Cohen S., Lippincott-Schwartz J. (2015). Fatty acid trafficking in starved cells: regulation by lipid droplet lipolysis, autophagy, and mitochondrial fusion dynamics. Dev. Cell.

[48] Huang S.C., Everts B., Ivanova Y., O'Sullivan D., Nascimento M., Smith A.M. (2014). Cell-intrinsic lysosomal lipolysis is essential for alternative activation of macrophages. Nat. Immunol..

[49] Vogel A., Brunner J.S., Hajto A., Sharif O., Schabbauer G. (2022). Lipid scavenging macrophages and inflammation. Biochim. Biophys. Acta Mol. Cell Biol Lipids.

[50] Ouimet M., Franklin V., Mak E., Liao X., Tabas I., Marcel Y.L. (2011). Autophagy regulates cholesterol efflux from macrophage foam cells via lysosomal acid lipase. Cell Metab..

[51] Yang X., Lu X., Lombes M., Rha G.B., Chi Y.I., Guerin T.M. (2010). The G(0)/G(1) switch gene 2 regulates adipose lipolysis through association with adipose triglyceride lipase. Cell Metab..

[52] Cohen-Kaplan V., Livneh I., Avni N., Fabre B., Ziv T., Kwon Y.T. (2016). p62- and ubiquitin-dependent stress-induced autophagy of the mammalian 26S proteasome. Proc. Natl. Acad. Sci. U. S. A..

[53] Padmanabha Das K.M., Wechselberger L., Liziczai M., De la Rosa Rodriguez M., Grabner G.F., Heier C. (2018). Hypoxia-inducible lipid droplet-associated protein inhibits adipose triglyceride lipase. J. Lipid Res..

[54] Schott M.B., Rasineni K., Weller S.G., Schulze R.J., Sletten A.C., Casey C.A. (2017). beta-Adrenergic induction of lipolysis in hepatocytes is inhibited by ethanol exposure. J. Biol. Chem..

[55] Camell C.D., Sander J., Spadaro O., Lee A., Nguyen K.Y., Wing A. (2017). Inflammasome-driven catecholamine catabolism in macrophages blunts lipolysis during ageing. Nature.

[56] Valm A.M., Cohen S., Legant W.R., Melunis J., Hershberg U., Wait E. (2017). Applying systems-level spectral imaging and analysis to reveal the organelle interactome. Nature.

[57] Zimmermann J.A., Lucht K., Stecher M., Badhan C., Glaser K.M., Epple M.W. (2024). Functional multi-organelle units control inflammatory lipid metabolism of macrophages. Nat. Cell Biol..

[58] Menon D., Bhapkar A., Manchandia B., Charak G., Rathore S., Jha R.M. (2023). ARL8B mediates lipid droplet contact and delivery to lysosomes for lipid remobilization. Cell Rep..

[59] (2023). Spartin is a receptor for the autophagy of lipid droplets. Nat. Cell Biol..

[60] Coats B.R., Schoenfelt K.Q., Barbosa-Lorenzi V.C., Peris E., Cui C., Hoffman A. (2017). Metabolically activated adipose tissue macrophages perform detrimental and beneficial functions during diet-induced obesity. Cell Rep..

[61] Lee Y.H., Petkova A.P., Granneman J.G. (2013). Identification of an adipogenic niche for adipose tissue remodeling and restoration. Cell Metab..

[62] Stansbury C.M., Dotson G.A., Pugh H., Rehemtulla A., Rajapakse I., Muir L.A. (2023). A lipid-associated macrophage lineage rewires the spatial landscape of adipose tissue in early obesity. JCI Insight..

[63] Chang S.C., Ding J.L. (2014). Ubiquitination by SAG regulates macrophage survival/death and immune response during infection. Cell Death Differ.

[64] Ma B., Cheng H., Mu C., Geng G., Zhao T., Luo Q. (2019). The SIAH2-NRF1 axis spatially regulates tumor microenvironment remodeling for tumor progression. Nat. Commun..

[65] Shapiro H., Pecht T., Shaco-Levy R., Harman-Boehm I., Kirshtein B., Kuperman Y. (2013). Adipose tissue foam cells are present in human obesity. J. Clin. Endocrinol. Metab..

[66] Cochain C., Vafadarnejad E., Arampatzi P., Pelisek J., Winkels H., Ley K. (2018). Single-cell RNA-Seq reveals the transcriptional landscape and heterogeneity of aortic macrophages in murine atherosclerosis. Circ. Res..

[67] Zhang X., Wang B., Wang C., Chen L., Xiao Y. (2015). Monitoring lipid peroxidation within foam cells by lysosome-targetable and ratiometric probe. Anal Chem..

[68] Jarc E., Petan T. (2019). Lipid droplets and the management of cellular stress yale. J. Biol. Med..

[69] Emanuel R., Sergin I., Bhattacharya S., Turner J., Epelman S., Settembre C. (2014). Induction of lysosomal biogenesis in atherosclerotic macrophages can rescue lipid-induced lysosomal dysfunction and downstream sequelae. Arterioscler Thromb. Vasc. Biol..

[70] Mekbib T., Suen T.C., Rollins-Hairston A., Smith K., Armstrong A., Gray C. (2022). The ubiquitin ligase SIAH2 is a female-specific regulator of circadian rhythms and metabolism. PLoS Genet..

[71] Ghosh S., Taylor J.L., Mendoza T.M., Dang T., Burk D.H., Yu Y. (2019). Siah2 modulates sex-dependent metabolic and inflammatory responses in adipose tissue to a high-fat diet challenge. Biol. Sex Differ.

[72] Qi J., Tripathi M., Mishra R., Sahgal N., Fazli L., Ettinger S. (2013). The E3 ubiquitin ligase Siah2 contributes to castration-resistant prostate cancer by regulation of androgen receptor transcriptional activity. Cancer Cell.

[73] Walczak R., Tontonoz P. (2002). PPARadigms and PPARadoxes: expanding roles for PPARgamma in the control of lipid metabolism. J. Lipid Res..

[74] Wang Z., Wang M., Xu X., Liu Y., Chen Q., Wu B. (2023). PPARs/macrophages: a bridge between the inflammatory response and lipid metabolism in autoimmune diseases. Biochem. Biophys. Res. Commun..

[75] Metzger M.B., Pruneda J.N., Klevit R.E., Weissman A.M. (2014). RING-type E3 ligases: master manipulators of E2 ubiquitin-conjugating enzymes and ubiquitination. Biochim. Biophys. Acta.

[76] Qi J., Nakayama K., Gaitonde S., Goydos J.S., Krajewski S., Eroshkin A. (2008). The ubiquitin ligase Siah2 regulates tumorigenesis and metastasis by HIF-dependent and -independent pathways. Proc. Natl. Acad. Sci. U. S. A..

[77] Oppi S., Nusser-Stein S., Blyszczuk P., Wang X., Jomard A., Marzolla V. (2020). Macrophage NCOR1 protects from atherosclerosis by repressing a pro-atherogenic PPARgamma signature. Eur. Heart J..

[78] Ricote M., Glass C.K. (2007). PPARs and molecular mechanisms of transrepression. Biochim. Biophys. Acta.

[79] Maridas D.E., Rendina-Ruedy E., Le P.T., Rosen C.J. (2018). Isolation, culture, and differentiation of bone marrow stromal cells and osteoclast progenitors from mice. J. Vis. Exp..

[80] Able A.A., Richard A.J., Stephens J.M. (2018). Loss of DBC1 (CCAR2) affects TNFalpha-induced lipolysis and Glut4 gene expression in murine adipocytes. J. Mol. Endocrinol..

