# Supplementary material for: Siah2 regulates lipid uptake in adipose tissue macrophages
Source: J Biol Chem. 2026 Mar 18;302(5):111380. doi: 10.1016/j.jbc.2026.111380 (PMC13090517; doi:10.1016/j.jbc.2026.111380)
Supplement: Supplementary Material — 1 [file mmc1.pdf]

Siah2 Regulates Lipid Uptake in Adipose Tissue Macrophages

Bhaswati Ghosh, Pradip R. Panta, Matthew C. Scott, Jessica Taylor, Robbie Beyl, Krisztian Stadler, Z. Elizabeth Floyd

List of material **page**

Fig. S1 related to Figure 1	1
Fig. S2 related to Figure 2	2
Fig. S3 related to Figure 4	3
Fig. S4 related to Figure 6	4
Fig. S5 related to Figure 8	5
Table S1	6
Table S2	9

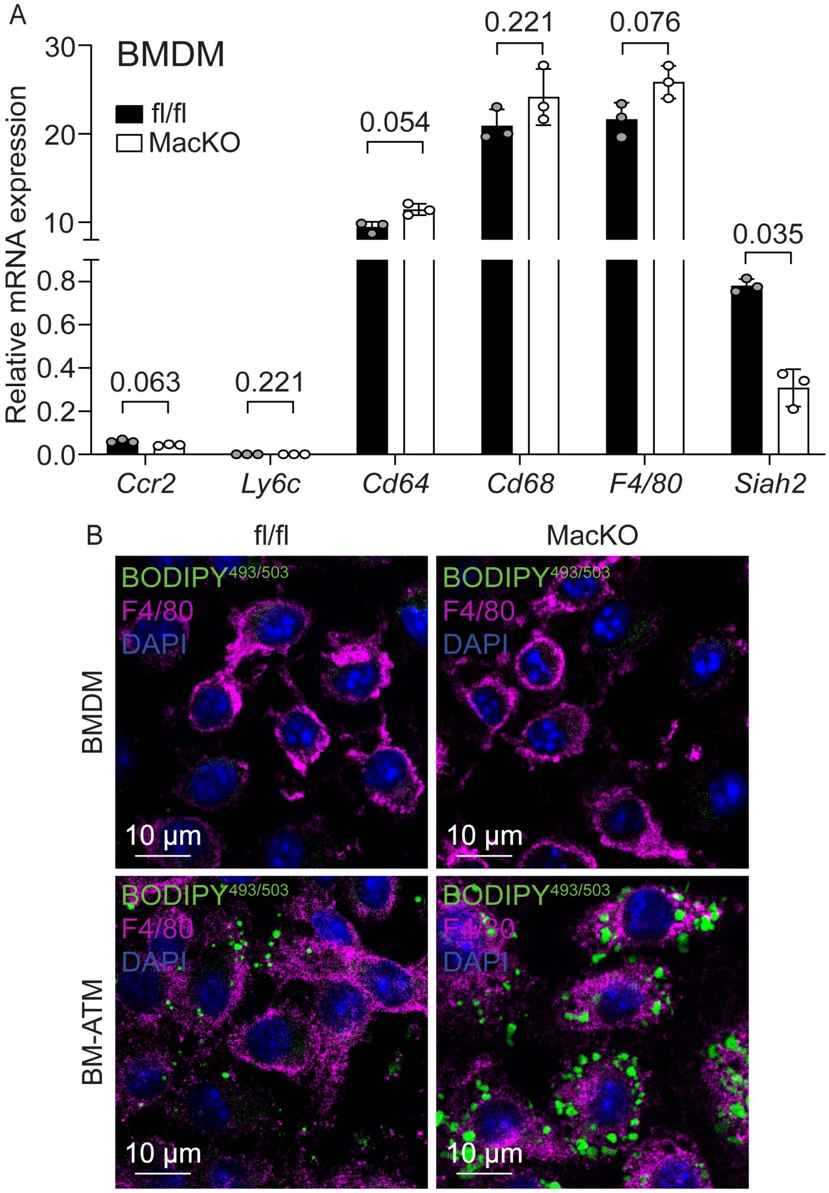

Fig. S1 related to Figure. 1. Macrophage-specific Siah2 loss results in lipid accumulation in ATMs. (A) Relative mRNA expression of monocyte markers (*Ccr2, Ly6c*), macrophage markers (*Cd64, Cd68, F4/80*) and *Siah2* in BMDMs derived from fl/fl and *Siah2*^{MacKO} mice. Statistics are reported as mean ± SD using unpaired t test with Welch's correction. Data are representative of three independent experiments. *p* values are indicated on the graphs (B) Representative confocal images of fl/fl and *Siah2*^{MacKO} BMDMs and BM-ATMs, immunolabelled for F4/80 (macrophage, magenta) and stained for BODIPY$^{493/503}$ (LD, green) and DAPI (nuclei, blue). fl/fl, *Siah2*$^{flox/flox}$; MacKO, *Siah2*^{MacKO}

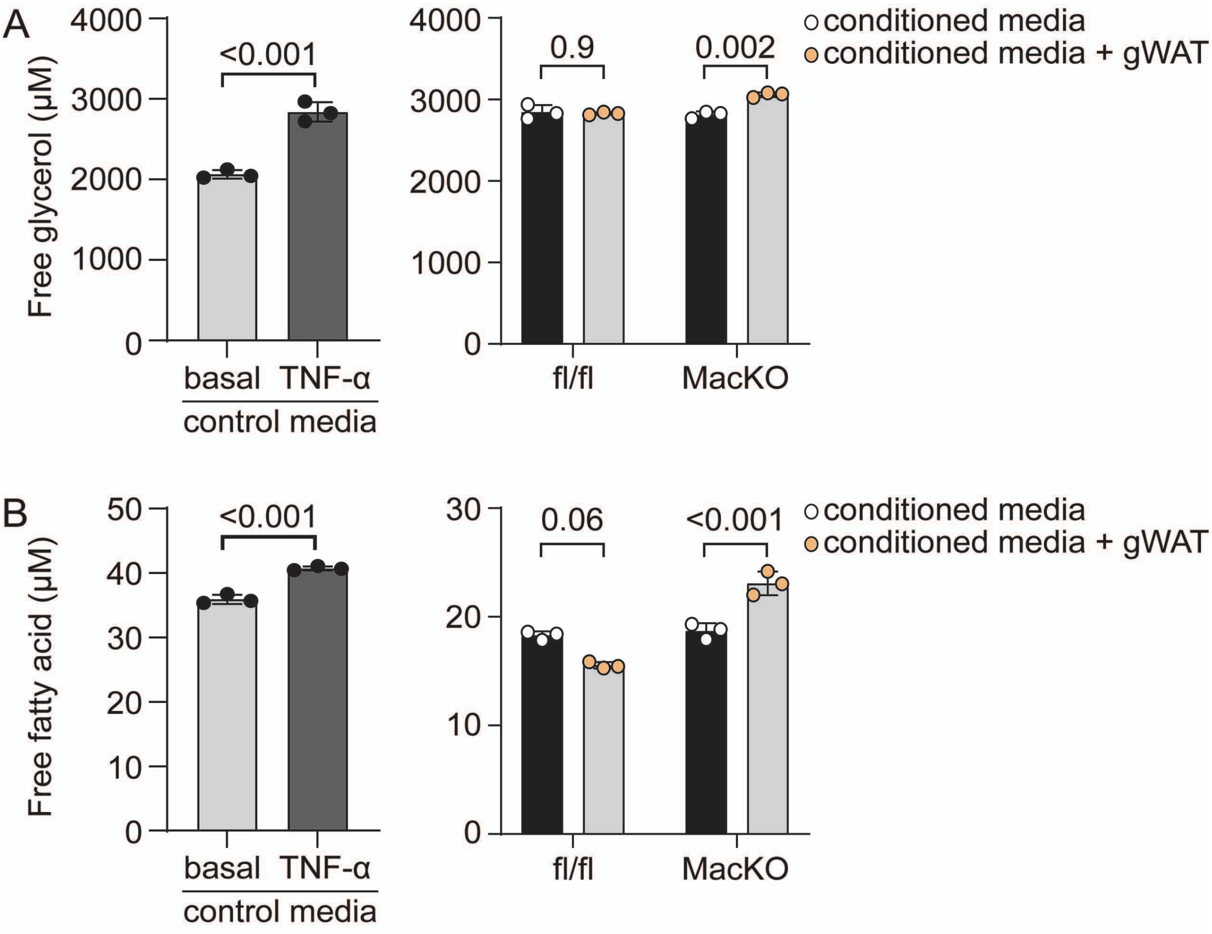

Fig. S2 related to Fig. 2. Increased inflammation in Siah2-deficient adipose tissue macrophages induces adipose tissue lipolysis. (A, left) Glycerol release in gWAT treated with vehicle or TNF-α. (A, right) Black bars represent glycerol release in fl/fl and *Siah2*^{MacKO} BM-ATM conditioned media. Gray bars represent glycerol release in adipose tissue treated with fl/fl and *Siah2*^{MacKO} BM-ATM conditioned media. (B, left) Free fatty acid release in gWAT treated with vehicle or TNF-α. (B, right) Black bars represent free fatty acid release in fl/fl and *Siah2*^{MacKO} BM-ATM conditioned media. Gray bars represent free fatty acid release in adipose tissue treated with fl/fl and *Siah2*^{MacKO} BM-ATM conditioned media. Statistics are reported as mean ± SD using unpaired t test with Welch's correction. Each dot denotes technical replicates representative of two independent experiments. *p* values are indicated on the graphs. fl/fl, *Siah2*$^{flox/flox}$; MacKO, *Siah2*^{MacKO}

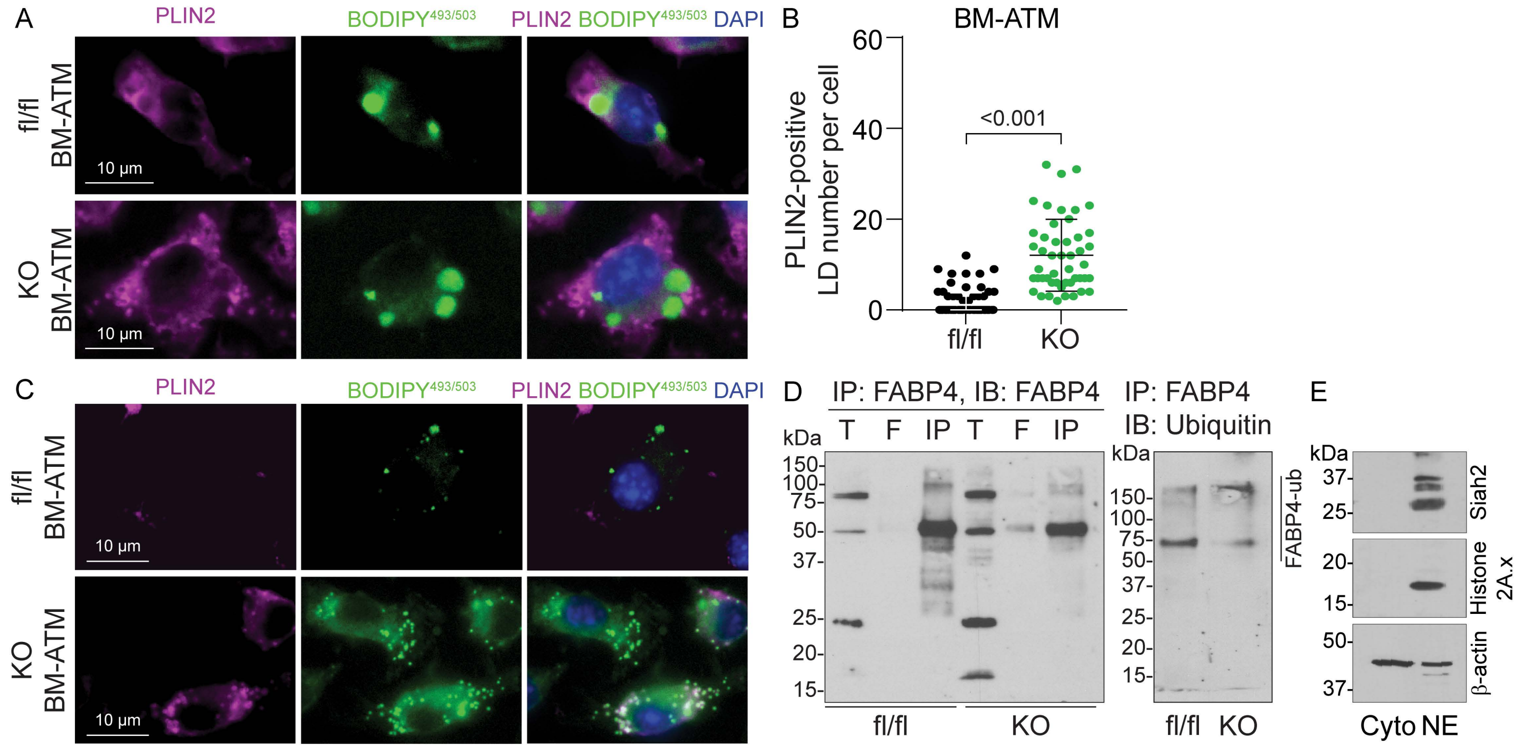

Fig. S3 related to Fig. 4. Siah2 depletion in ATMs increases PLIN2-positive LD abundance. (A) fl/fl and *Siah2*^{MacKO} BM-ATMs, immunolabeled for PLIN2 (magenta), stained for BODIPY$^{493/503}$ (LD, green) and DAPI (nuclei, blue). Scale bar 10 µm. (B) Quantification of PLIN2-positive LD number per cell in fl/fl and *Siah2*^{MacKO} BM-ATMs shown in A. (C) fl/fl BM-ATMs, immunolabeled for PLIN2 (magenta), stained for BODIPY$^{493/503}$ (LD, green) and DAPI (nuclei, blue). Red signals were converted to magenta using the LUT function in ImageJ. Scale bar 10 µm. (D) fl/fl and *Siah2*^{MacKO} BM-ATM whole cell extracts were immunoprecipitated using anti-FABP4 antibody and subjected to western blot analyses using either anti-FABP4 antibody (IP: FABP4, IB: FABP4) or anti-ubiquitin antibody (IP: FABP4, IB: Ubiquitin). Western blot images are representative of two independent experiments. T, total lysate; F, flow-through; IP, immunoprecipitation. (E) fl/fl BM-ATMs were treated with 20 nM leptomycin B for 30 minutes prior to harvesting. Nuclear fraction (NE) and cytoplasmic fraction (Cyto) were extracted and subjected to western blot analyses using anti-SIAH2 antibody. Histone 2A.x marked the nuclear fraction. β-actin was used as loading control, present in both cytoplasmic and nuclear fractions. Statistics are reported as mean ± SD using unpaired t test with Welch's correction. *n*=3 male mice per group. *p* values are indicated on the graphs. fl/fl, *Siah2*$^{flox/flox}$; KO, Siah2^{MacKO}

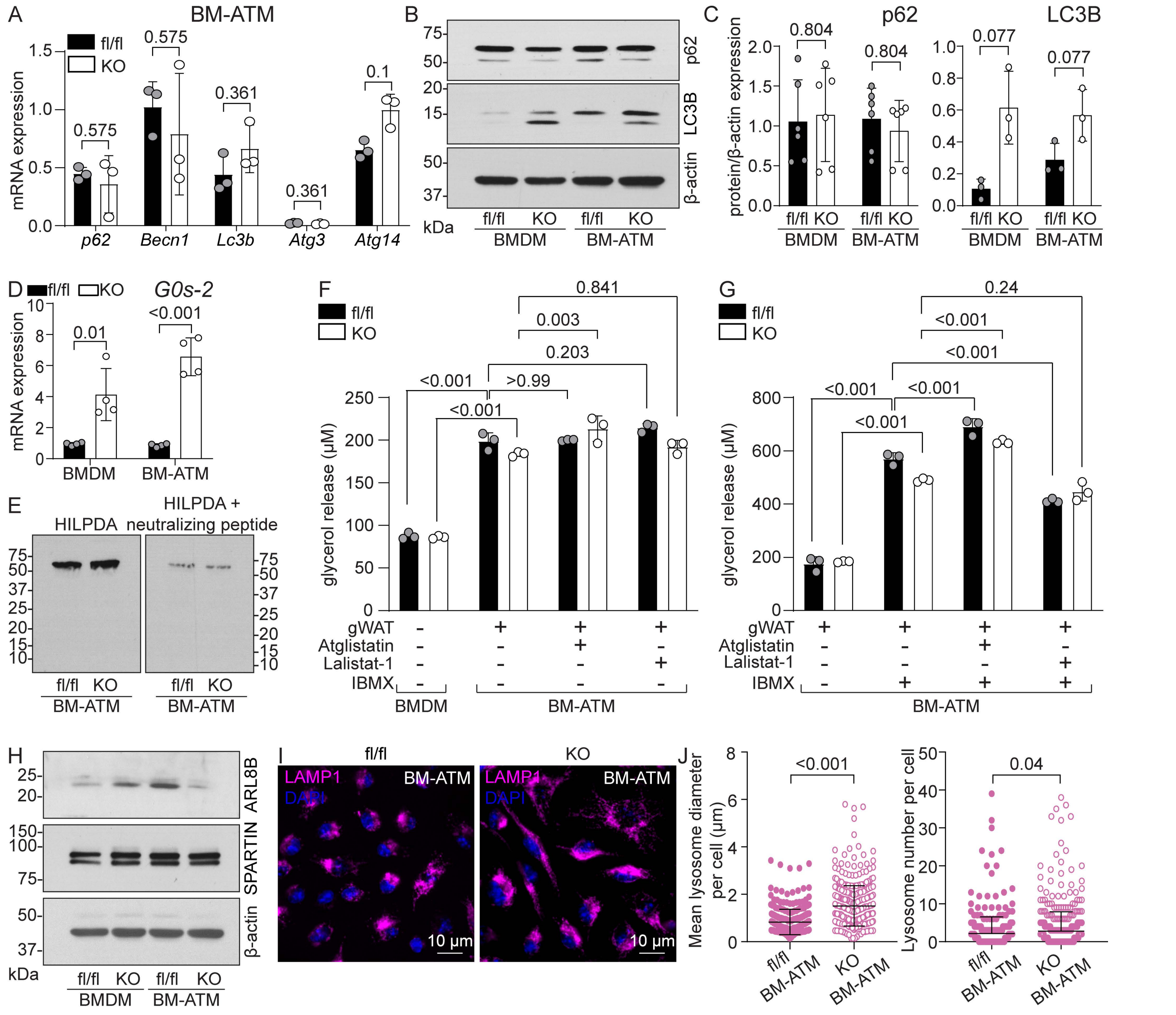

Fig. S4 related to Fig. 6. Siah2 does not influence autophagy or ATGL- and LAL-mediated lipolysis in ATMs. (A) Relative mRNA expression of autophagy genes in fl/fl and *Siah2*^{MacKO} BMATMs. (B-C) Western blot (B) and quantification (C) of p62 and LC3B protein levels relative to β-actin in fl/fl and *Siah2*^{MacKO} BMDMs and BM-ATMs. (D) Relative mRNA expression of *G0s2* in fl/fl and *Siah2*^{MacKO} BMDM and BM-ATMs. (E) Western blot for HILPDA protein levels with and without neutralization in fl/fl and *Siah2*^{MacKO} BM-ATMs. (F) Two-way ANOVA analyses of Figure 6G. (G) Two-way ANOVA analyses of Figure 6H. (H) Representative western blot for ARL8B and SPARTIN protein levels relative to β-actin in fl/fl and *Siah2*^{MacKO} BMDMs and BM-ATMs. The β-actin panel is same as used in Figure 4H. The blot was reprobed for the images in Figure S4H. (I) Representative fluorescence microscopy images of fl/fl and *Siah2*^{MacKO} BM-ATMs immunolabelled for LAMP1 (lysosomes, magenta) and stained for DAPI (nuclei, blue). Red signals of LAMP1 were converted to magenta using the LUT function in ImageJ. Scale bars 10 µm. (J) Quantification of Figure 6I. Mean lysosome diameter (µm) per cell and lysosome number per cell in fl/fl and *Siah2*^{MacKO} BM-ATMs. Statistics are reported as mean ± SD using unpaired t test with Welch's correction (A, C, D and J) and two-way ANOVA with Tukey's multiple comparisons test (F and G). *n*=3-6 male mice per group and are representative of 3 independent experiments. Each dot represents technical replicates. *p* values are indicated on the graphs. fl/fl, *Siah2*$^{flox/flox}$; MacKO, *Siah2*^{MacKO}

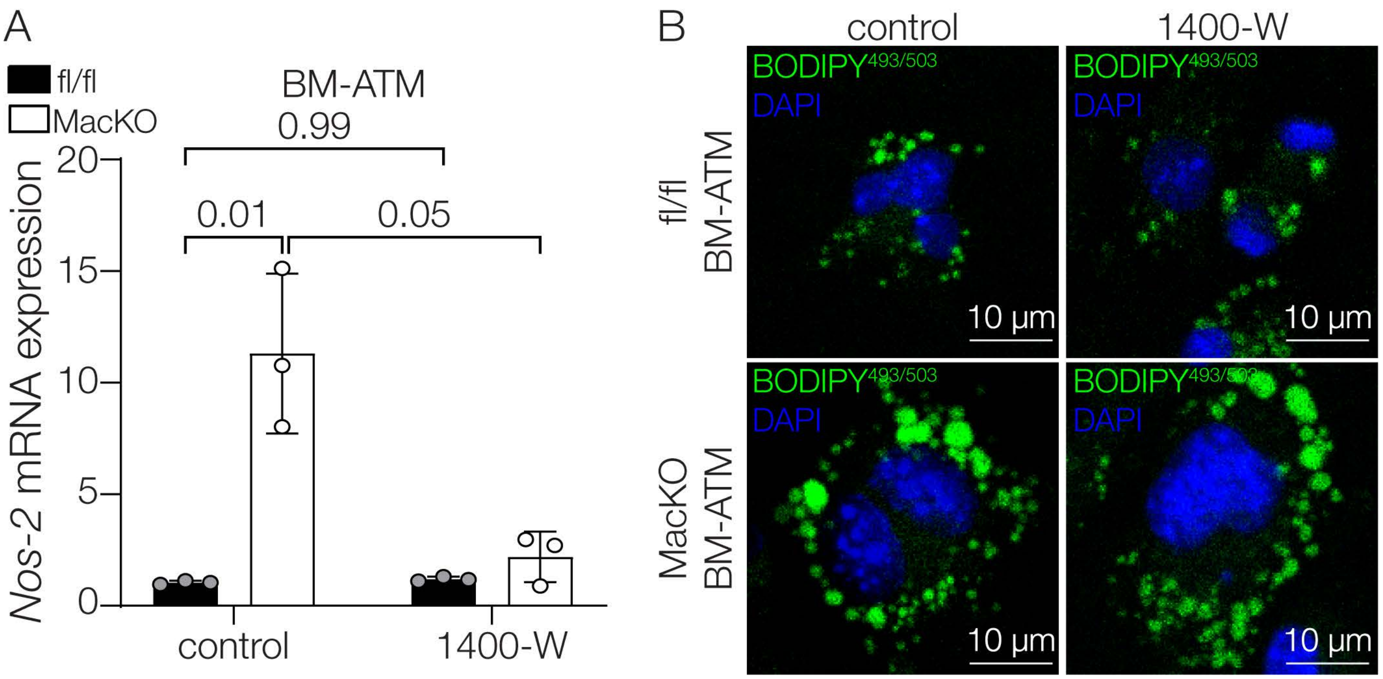

Fig. S5 related to Figure 8. iNOS inhibition does not alter lipid accumulation in BM-ATMs.

(A) Relative mRNA expression of *Nos-2* in fl/fl and *Siah2*^{MacKO} BM-ATMs in the presence or absence of 1400-W (iNOS inhibitor). (B) Confocal images of fl/fl and *Siah2*^{MacKO} BM-ATMs stained for BODIPY$^{493/503}$ (LD, green) and DAPI (nuclei, blue) in the presence or absence of 1400-W. Scale bars 10 μm. Statistics are reported as mean ± SD using two-way ANOVA with Tukey's multiple comparisons test. Each dot represents technical replicates. *p* values are indicated on the graphs. fl/fl, *Siah2*$^{flox/flox}$; MacKO, *Siah2*^{MacKO}

Table S1. A list of primers used in this study for RT-PCR

Gene symbol	Gene name	Forward sequence	Reverse sequence
18S	18S ribosomal RNA	5'-AGT CCC TGC CCT TTG TAC ACA	5'-GAT CCG AGG GCC TCA CTA AAC
Siah2	Seven in Absentia Homology 2	5'-ACC AGA GCA TGA AGA CAT CTG TGA	5'-TGG GCA TGC ATG AGA TGG GA
Cd36	Cluster of Differentiation 36	5'-GAA GCA TTA AAG AAT CTG AAG AGA AGA CC	5'-GGA TTT GCA AGC ACA ATA TGA AAT C
Trem2	Triggering Receptor Expressed on Myeloid cells 2	5'-GTC ACC ATC ACT CTG AAG AAC C	5'-GAG GTG CTG TGT TCC ACT T
Nos2	Inducible Nitric Oxide Synthase	5'-AGG AGG AGA GAG ATC CGA TTT AG	5'-TCC AAG GTG CTT GCC TTA TAC
Il10	Interleukin-10	5'-CTA TGC TGC CTG CTC TTA CTG	5'-CTC CAC TGC CTT GCT CTT ATT
Sqstm1	Sequestosome1	5'-GTG GTG GGA ACT CGC TAT AAG	5'-ATC TGG GAG AGG GAC TCA AT
Lc3B	Microtubule Associated Protein 1 Light Chain 3B	5'-CCA CCA AGA TCC CAG TGA TTA T	5'-CTA CAA CAC CAG ACC TGC TTA G
Becn1	Beclin1	5'-CAG GAA CTC ACA GCT CCA TTA C	5'-CTG CTC ACT GTC ATC CTC ATT C
Atg3	Autophagy Related 3	5'-CCG GTC CTC AAG GAA TCA AA	5'-GAC TCT ATA GCC TCT CCA CTA CA
Atg14	Autophagy Related 14	5'-GAG CTC ACC TCC ATC ATA TTC C	5'-ACT CCA GGG TCC ACA AAT TC
Saa3	Serum Amyloid A-3	5'-GCC TTC CAT TGC CAT CAT TC	5'-CAC ATG TCT CTA GAC CCT TGA C
Gdf3	Growth Differentiation Factor 3	5'-CAG GAC TTA TGC TAC GTG AAG G	5'-CTT GGA AAG GTT TCT GTG TAT TAA GG
Tnfα	Tumor Necrosis Factor alpha	5'-CCT CTT CTC ATT CCT GCT TGT	5'-TGG GAA CTT CTC ATC CCT TTG
Pparg1	Peroxisome Proliferator-Activated Receptor Gamma 1	5'-GCG GTG AAC CAC TGA TAT TC	5'-CTG GAG AAA TCA ACT GTG GTA AAG
G0s2	G0/G1 Switch 2	5'-CAAAGCCAGTCTGACGCAA	5'-CTGCACACTTTCCATCTGA

Gene symbol	Gene name	Forward sequence	Reverse sequence
Plin2	Perilipin-2	5’-CAA CAG AGC GTG GTG ATG A	5’-CGG GTA CTG ATC CTT TGT ACT G
Ctsk	Cathepsin k	5’-TCC GCA ATC CTT ACC GAA TAA A	5’-CCA CAT CCT GCT GTT GAG AA
Atp6v0d2	V-type Proton ATPase Subunit d 2	5’-AGC CAG CCT AAC TCA GC	5’-GCT TCT TCC TCA TCT CCG TGT C
Il1β	Interleukin-1β	5’-CTC CAC CTC AAT GGA CAG AAT ATC	5’-TTG GGA TCC ACA CTC TCC A
Sr-a	Scavenger Receptor Class A	5’-AGG CGG ATC AAG ATC AGT ATA AC	5’-TGA CTT GTC CAG AGG TGA AAG
Lox1	Ox-LDL Receptor 1	5’-GAC TGG CTC TGG CAT AAA GAA A	5’-TGC TTG TAA GAT GAA TGT CAG ATC A
Cd206	Cluster of Differentiation 206	5’-GCC TTT GTT GAT GTC CCT AAT G	5’-CTT CCA ACT GCC AGA CTG T
Tfeb	Transcription Factor EB	5’-CCC GAG AAA GAG TTT GAT GAT G	5’-GTT CAG GTG GCT GCT AGA
Lipa	Lysosomal Acid Lipase	5’-CCC TCT TCT CAA GGA CAT GTT T	5’-GAA GCC ACA TAG CAG GAA GAA
Atgl	Adipose Triglyceride Lipase	5’-CTC ATA AAG TGG CAA GTT GTC TG	5’-GAG CTC ATC CAG GCC AAT
Igf1	Insulin-like growth factor1	5’-TAC TTC AAC AAG CCC ACA GG	5’-TCT CCA GTC TCC TCA GAT CAC
Resistin	Resistin	5’-CCA GAA GGC ACA GCA GTC	5’-GTC CAG TCT ATC CTT GCA CAC
Cxcl1	C-X-C motif chemokine ligand 1	5’-GGG ACA CCT TTT AGC ATC TTT TG	5’-ATG GCT GGG ATT CAC CTC AAG
Ccl2	CC motif chemokine ligand 2	5’-GCT CAG CCA GAT GCA GTT A	5’-CTG CTG GTG ATC CTC TTG TAG
Il6	Interleukin-6	5’-CTT CAC AAG TCG GAG GCT TAA T	5’-AAT TGC CAT TGC ACA ACT CTT T
F4/80	Adhesion G protein-coupled receptor E1	5’-AAC TCA AGG ACA CGA GGT TG	5’-GGT GAA CAG GTA TGC CAT GA
Ccr2	CC motif chemokine receptor 2	5’-CAG TTC ATC CAC GGC ATA CT	5’-TGA CAA GGC TCA CCA TCA TC

Gene symbol	Gene name	Forward sequence	Reverse sequence
Ly6c	Lymphocyte antigen 6	5'-ATG GAC ACT TCT CAC ACT ACA AA	5'-GCA GTC CCT GAG CTC TTT C
Cd64	Cluster of Differentiation 64	5'-GGC GAA TAC AGG TGT CAG ATA G	5'-GCA GCC AAT CAT TGT GGA TTT
Cd68	Cluster of Differentiation 68	5'-GGG CTC TTG GGA ACT ACA	5'-CCC AAG CCT TTC TTC CAC
Hilpda	Hypoxia inducible Lipid Droplet-Associated	5'-TGC TGG GCA TCA TGT TGA CC	5'-TGA CCC CTC GTG ATC CAG G

Table S2. Antibodies used for western blotting, immunoprecipitation* and immunostaining#

Antibody	Source	Catalog #
Mouse anti-4-HNE	Abcam	ab48506
Rabbit anti-ARL8B	Proteintech	13049-1-AP
Rabbit anti-ATP6V0D2	Millipore Sigma	ABS1677
Rabbit anti-ATGL	Cell Signaling Technology	2138S
Rabbit anti-β-actin	GeneTex	GTX109639
Mouse anti-CD36	Santa Cruz Biotechnology	sc-7309
Rabbit anti-CD36*	Proteintech	18836-1-AP
Mouse anti-CTSK	Santa Cruz Biotechnology	sc-48353
Rat anti-F4-80#	Abcam	ab6640
Rabbit anti-FABP4	Cell Signaling Technology	2120
Rabbit anti-FABP4*	LS-Bio	LS-C171962
Mouse anti-HILPDA	Santa Cruz Biotechnology	sc-376704
Mouse anti-HILPDA neutralizing peptide	Santa Cruz Biotechnology	sc-376704 P
Rabbit anti-HISTONE H2AX	AbClonal	A11361
Mouse anti-LAMP1#	Invitrogen	14-1071-82
Rabbit anti-LC3B	GeneTex	GTX127375
Rabbit anti-p62	Cell Signaling Technology	5114S
Rabbit anti-PLIN2#	Proteintech	15294-1-AP
Mouse anti-PPAR-γ (E-8)	Santa Cruz Biotechnology	Sc-7273
Rabbit anti-PPAR-γ	Fortis Life Sciences	A304-461A
Rabbit anti-SIAH2	LS-Bio	LS-C112149
Rabbit anti-SIAH2#	Proteintech	12651-1-AP
Rabbit anti-SPARTIN	Proteintech	13791-1-AP
Mouse anti-TREM-2	Cell Signaling Technology	29715
Rabbit anti-UBIQUITIN	Proteintech	10201-2-AP
Goat anti-rabbit Alexa Fluor-488#	Molecular Probes	A11034
Goat anti-rabbit Alexa Fluor-568#	Invitrogen	A11036
Goat anti-rat Alexa Fluor-647#	Abcam	ab150167
Peroxidase AffiniPure® Goat Anti-Rabbit IgG (H+L)	Jackson ImmunoResearch	111-035-003
Peroxidase AffiniPure® Goat Anti-Mouse IgG (H+L)	Jackson ImmunoResearch	115-035-003
